# Rapid riparian ecosystem decline in Rocky Mountain National Park

**DOI:** 10.1111/cobi.70053

**Published:** 2025-05-31

**Authors:** David J. Cooper, E. William Schweiger, Jeremy R. Shaw, Cherie J. Westbrook, Kristen Kaczynski, Hanem Abouelezz, Scott M. Esser, Koren Nydick, Isabel de Silva, Rodney A. Chimner

**Affiliations:** ^1^ Department of Forest and Rangeland Stewardship Colorado State University Fort Collins Colorado USA; ^2^ National Park Service Rocky Mountain Inventory and Monitoring Network Fort Collins Colorado USA; ^3^ Department of Geography and Planning and Centre for Hydrology University of Saskatchewan Saskatoon Saskatchewan Canada; ^4^ Department of Earth and Environmental Sciences California State University Chico California USA; ^5^ National Park Service Natural Resource Stewardship and Science Directorate Fort Collins Colorado USA; ^6^ National Park Service Rocky Mountain National Park Estes Park Colorado USA; ^7^ College of Forest Resources and Environmental Science Michigan Technological University Houghton Michigan USA

**Keywords:** alternative states, beaver, collapse, elk, herbivory, moose, riparian, willows, alce, castor, colapso, estados alternativos, herbivoría, ribereño, sauces

## Abstract

Understanding the drivers of ecosystem collapse is critical for resource management, particularly for protected areas mandated to preserve biodiversity. In Rocky Mountain National Park, Colorado, tall willows (*Salix* spp.) dominated riparian vegetation, and a beaver–willow state was the natural ecosystem type in the Colorado River headwaters. However, willows comprise a portion of elk diets and are a preferred food for recently introduced moose, and the vegetation structure has changed dramatically since the early 2000s. To assess ecosystem changes, we analyzed time‐series data on willow height from 1997 to 2021 inside and outside 3 exclosures built to exclude ungulates, area of tall willows in 1999 and 2019, area of open water from 1953 to 2019, vegetation composition in 1998 and 2021, groundwater depth from 1996 to 2021, surface water flow from 1953 to 2023, and climate from 1950 to 2023. Tall willow coverage and open water area declined by >90% from 1999 to 2019. Willow height outside the ungulate exclosures declined by more than 75% since the 1990s; yet, within exclosures that were formerly browsed, willow height increased by up to 500%. Tall willow communities have largely been replaced by grasslands. Browsing by elk and moose likely played a pivotal role in triggering a collapse of the beaver–willow state and the formation of an alternative moose–elk–grassland state that appears stable and may be difficult to reverse without direct human action. Restoration efforts will depend on a reduction in herbivory and reconnection of the river with its floodplain.

## Abstract (es)

Declinación acelerada de los ecosistemas ribereños en el Parque Nacional de las Montañas Rocallosas

**Resumen:**

Comprender los factores que provocan el colapso de los ecosistemas es fundamental para la gestión de los recursos, sobre todo en las zonas protegidas cuyo mandato es preservar la biodiversidad. En el Parque Nacional de las Montañas Rocallosas (Colorado), los sauces altos (*Salix* spp.) dominaban la vegetación ribereña y la relación castor‐sauce era el tipo de ecosistema natural en la cabecera del río Colorado. Sin embargo, los sauces constituyen una parte de la dieta de los alces y son el alimento preferido de los alces introducidos recientemente y la estructura de la vegetación ha cambiado drásticamente desde principios de la década de 2000. Para evaluar los cambios en el ecosistema, analizamos series temporales de datos sobre la altura de los sauces entre 1997 y 2021 dentro y fuera de tres cercados construidos para excluir ungulados, la superficie de sauces altos en 1999 y 2019, la superficie de aguas abiertas entre 1953 y 2019, la composición de la vegetación en 1998 y 2021, la profundidad de las aguas subterráneas entre 1996 y 2021, el caudal de aguas superficiales entre 1953 y 2023, y el clima entre 1950 y 2023. La cobertura de sauces altos y la superficie de aguas abiertas disminuyeron en >90% de 1999 a 2019. La altura de los sauces fuera de los cercados de ungulados ha disminuido en más de un 75% desde la década de 1990; sin embargo, dentro de los cercados en los que antes se ramoneaba, la altura de los sauces ha aumentado hasta en un 500%. Las comunidades de sauces altos han sido sustituidas en gran medida por praderas. Es probable que el pastoreo de alces haya desempeñado un papel fundamental en el colapso de la relación castor‐sauce y la formación de una relación alternativa alce‐pastizal que parece estable y puede ser difícil de revertir sin la acción directa del hombre. Los esfuerzos de restauración deberían incluir la reducción de la herbivoría y la reconexión del río con su llanura aluvial.

## INTRODUCTION

Ecosystem collapse is the rapid, and sometimes abrupt, degradation of ecosystem biodiversity, functions, and services (Bergstrom et al., 2021; Bland et al., 2018; Keith et al., 2023; Newton et al., 2021). It is characterized by substantial and lasting loss or displacement of biota and reorganization of ecosystem structure and ecological processes (Keith et al., 2023). Predicting ecosystem collapse is a key challenge in ecology, and understanding and averting a collapse provides a foundation for effective conservation and management (Bergstrom et al., 2021). An ecosystem collapse may lead to the formation of alternate ecological states with changes in biotic and abiotic components, such as the loss of key species or communities (Holling, 1973; Scheffer & Carpenter, 2003). Collapse can lead to hysteresis, where more than one stable state can occur with conditions that prevent or restrict return to the original state (Suding & Gross, 2006). Identifying likely leading indicators of collapse often begins by describing an initial state based on multiple lines of evidence when possible (Bland et al., 2018). Time‐series data of key drivers and ecological responses are important for identifying the timing, patterns, and processes of change. Understanding how to predict ecosystem changes that signal an impending collapse (Sato & Lindenmayer, 2018) could inform management decisions that might avert or potentially reverse a collapse by overcoming the forces of hysteresis.

Among the most ecologically and socially valuable ecosystem types in semiarid and arid regions of the world are wetland and riparian areas. They depend on inputs of surface and groundwater from their watershed and support highly productive vegetation, with vital habitat for migratory birds, resident amphibians, and small and large mammals, and are often hotspots of biodiversity (Schweiger et al., 2016). Yet, many waterways are regulated by human‐made dams, water diversions, and groundwater extraction that can trigger ecosystem change and collapse (Merritt & Cooper, 2000; Rood & Mahoney, 1990). Above 2500‐m elevation in the Rocky Mountains, willows (*Salix* spp.) dominate riparian vegetation composition, structure, and biomass providing food and wood for beavers (*Castor canadensis*) that build dams ponding and redistributing water and sediment (Westbrook et al., 2011). Beavers maintain ecosystem functions, such as stream and floodplain interactions (Schweiger et al., 2016; Westbrook et al., 2006), willow establishment and growth (Cooper et al., 2006a; Woods & Cooper, 2005), and resistance to warm temperatures, droughts, floods, wildfires, and other factors amplified by climate change (Jordan & Fairfax, 2022). Engineering by beavers links streams and their aquatic environments with terrestrial riparian zones.

Most national parks in the United States and globally were created for the protection of biological diversity, landscape features, or historic sites (Dudley & Stolton, 2016). However, not all ecological or hydrologic processes are within the management domain of a park (Cole et al., 2008; Hobbs et al., 2010). For example, the movement of non‐native plants or animals into parks cannot always be controlled, nor can changes in climate, the introduction of diseases, or regulated river flows in managed watersheds. Many national parks, including Rocky Mountain National Park (RMNP), were established long after significant landscape alterations had already occurred. Park managers often try to influence complex processes—sometimes by purposefully not acting. For example, in 1968, Yellowstone National Park and RMNP adopted natural regulation policy decisions allowing natural processes to regulate ungulate populations even though apex predators had been reduced or extirpated (Huff & Varley, 1999). This policy was adopted after decades of using lethal methods to control ungulate populations (Baker et al., 1997).

**FIGURE 1:**
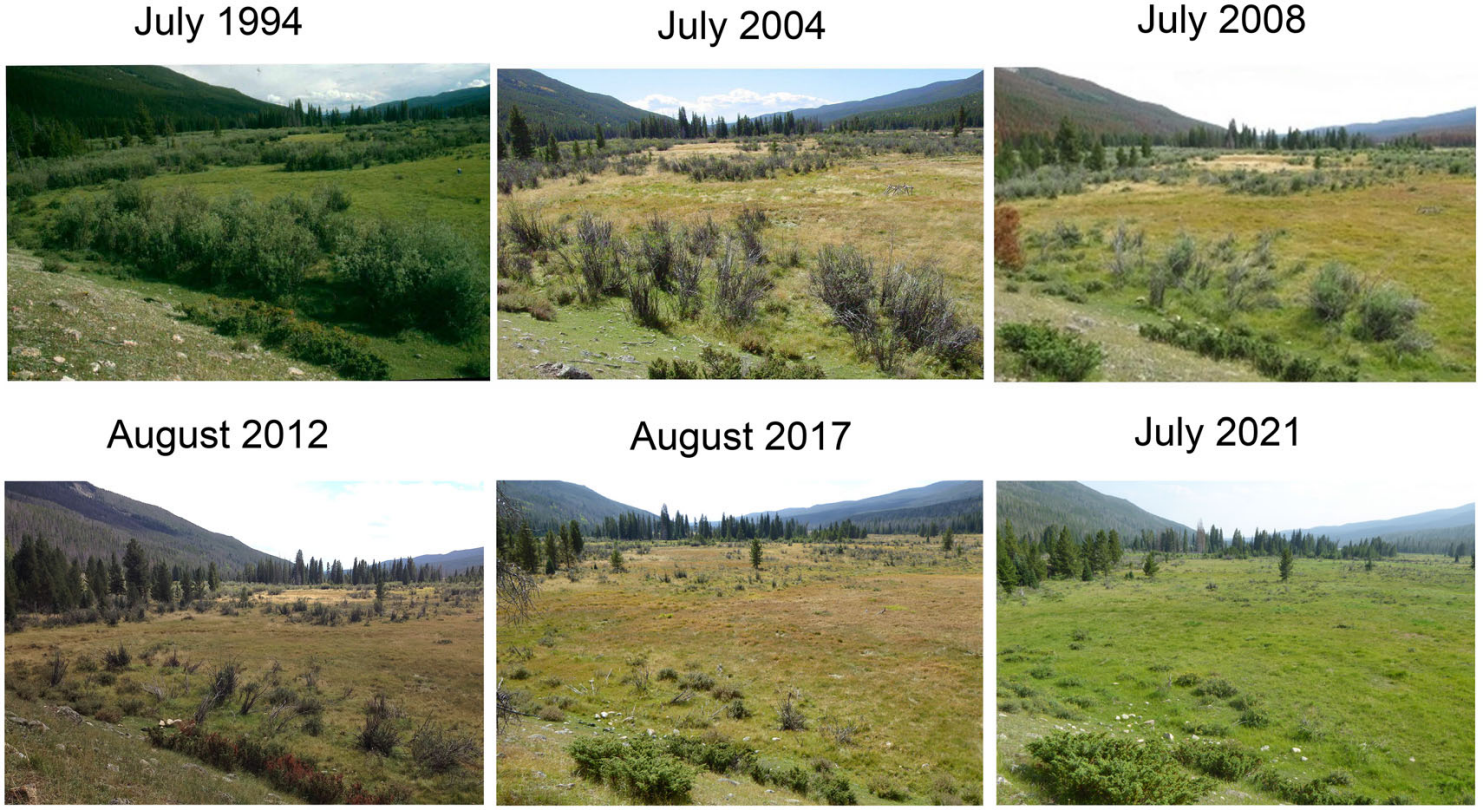
Photo sequence of tall willow collapse from 1994 to 2021 in the Kawuneeche Valley, Rocky Mountain National Park, Colorado (USA). See Figure 2 for location where the photos were taken. All photos by D.J. Cooper.

A time series of ground photos taken of the Colorado River headwaters in RMNP from 1994 to 2021 illustrates a dramatic change in vegetation structure (Figure 1). The ecological change occurred in the Colorado River headwaters in RMNP, known as the Kawuneeche Valley (KV) (Andrews, 2015), and is noteworthy not only because it occurred in a national park but also because it occurred over a relatively short period (30–40 years) in a system that had experienced many other stressors but had remained resilient until the modern period. We identified what processes led to the collapse of Colorado River riparian ecosystems in RMNP and what recovery pathways are possible. We used multiple lines of evidence to constrain the timing of the loss of the initial beaver–willow state and the establishment of a moose–elk–grassland state and to evaluate possible drivers of the collapse, including ungulate herbivory, loss of beaver, changes in climate, and competing mandates in the management of protected areas. We also considered management options for restoring the beaver–willow state.

## METHODS

### Study area

**FIGURE 2:**
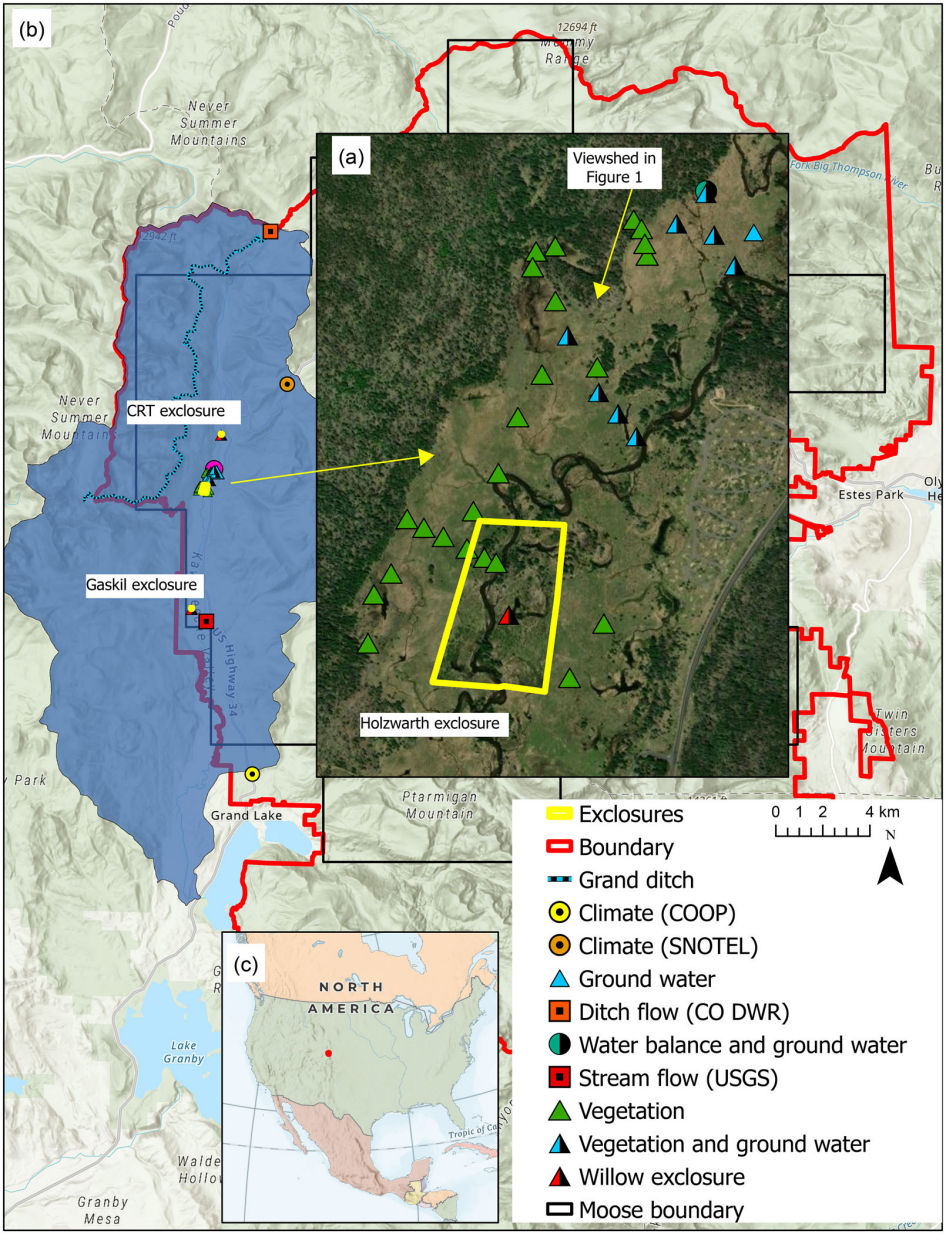
Sample locations for groundwater, vegetation, willow height (in and out of elk and moose exclosures), stream or ditch flow, climate, and water balance and of elk and moose exclosures in and around the Kawuneeche Valley, Rocky Mountain National Park, Colorado (USA): (a) enlargement of a sampling area, sampling sites and location of viewshed in Figure 1; (b) extent of the park (red), Kawuneeche watershed (blue), most recent moose study region (black; Abouelezz & Hobbs, 2025), and some sampling sites that are not visible in (a) (some portions of boundaries are overlaid by other map elements); and (c) location of the park (red dot) in North America (CRT, Colorado River trailhead).

All vegetation, water, and climate data were from the KV, were retrospective, and were assembled from long‐term studies spanning 1950 to 2023. Ungulate data either spatially include the KV or, in the case of elk, have a nexus to the KV, based on seasonal migrations and space use. The KV is 14 km^2^ with a watershed area of 138 km^2^ spanning 2667 to 3944‐m elevation (Figure 2). Valley bottom vegetation circa 1990 was a mix of wetland types including tall willow riparian shrublands, marshes, and fens (Chimner & Cooper, 2003b). Elk have been present in the southern Rocky Mountains through recorded time, with a brief period when they were extirpated from the area due to excess hunting. Moose were rarely present in Colorado's recorded history (Armstrong, 1987) but were introduced in 1978–1979 just 25 km west of the KV (Yost, 2008). Moose were first observed in the KV in 1980 (Stevens, 1988) and now use the valley and surrounding forests year‐round (Zeigenfuss & Abouelezz, 2018). Additional study area details are in Appendix S1. Plant species nomenclature follows Weber and Wittmann (2012) and Ackerfield (2022). Data used in this paper are available at: https://doi.org/10.5061/dryad.2z34tmpvz.

### Climate and water balance

Daily air temperature and precipitation records were extracted from the Grand Lake 1NW Cooperative Observer Network (COOP) National Centers for Environmental Information (NCEI) station from 1950 to 2023. Daily snow water equivalent (SWE) was obtained from the Lake Irene snowpack telemetry (SNOTEL) station 565 from 1980 through 2023. We calculated a daily water balance (WB) (Lutz et al., 2010; Thoma et al., 2020) (Appendix S2) at a central location in the KV. WB variables included climatic deficit (amount by which potential evapotranspiration exceeds actual evapotranspiration) as an index of drought (Adler & Hostetler, 2013), potential evapotranspiration, and cumulative runoff (Croke et al., 2005). Climate and WB were summarized as monthly medians or totals for a May to September growing season (GS) and SWE as a week of April 1 maximum. GSs are treated as independent observations and used to help reduce seasonal variability. Medians accommodate data with outliers and nonnormal distributions, such as most climate variables. Temporal trends in climate and WB were estimated using mixed‐effects generalized additive models (GAMs) (Wood, 2011) (Appendix S2). A period of record (POR) trend was estimated as a linear term where possible. The models account for random within‐year and fixed across‐year variation, with nonlinear terms for day within GS and year. Models also include a test for longer term patterns via a Pacific Decadal Oscillation term (PDO) (NOAA, 2024; Zhang et al., 1997). Model selection was guided by Akaike information criterion (AIC) values with a threshold of delta 2.0.

### Surface water

We summarized daily Colorado River stream flow at Baker Gulch from 1953 through 2023 as GS monthly means. A 23‐km long transmountain water diversion, the Grand River Ditch, was constructed from 1890 to 1936 and can divert an annual average of 20,000,000 m^3^·year^−1^, which is 0.4–0.6 of the tributary flow of the Colorado River into the South Platte River headwaters (Schweiger et al., 2016; Woods, 2000). We compared GS flow in the River and Ditch to estimate the proportion of water diverted across our POR. The POR trend in mean Colorado River flow, the standard deviation of logged flow, and the proportion of river flow diverted were estimated using GAMs as described above and in Appendix S2.

The area of ponded water in the RMNP portion of the KV, primarily beaver ponds, was manually digitized at a reference scale of 1:1000 in GIS from georeferenced imagery for 1953, 1990, 1999, 2005, 2009, 2015, and 2019. We used a GAM with a single predictor for year to evaluate the trend in total pond area and mean pond size across these 7 years (Appendix S2).

### Groundwater

Multiple monitoring wells were installed in the KV in the 1990s (Chimner & Cooper, 2003a) and early 2000s (Westbrook et al., 2006) to measure water table depth. Depth to water from the ground surface (DTW) was measured manually on a weekly basis during the GS from the 1990s through 2005. In 2007, loggers that measured DTW hourly were installed in several KV wells (Schweiger et al., 2015). Ten wells, all in similar tall willow sites, in the 1990s provided comparable estimates of GS groundwater depths. The DTW for 2007–2023 was first averaged to daily values and then divided into subsets of every 7th day to match manual data from 1996 to 2005. The trend in DTW was modeled using a mixed‐effects GAM with sites, days within GS, and years treated as random nonlinear effects. We also tested for the effects of climatic deficit, Colorado River flow, and 1‐year lagged SWE as fixed terms. Finally, because several wells were near beaver dams from 1996 to 2002 or 2004, we also tested for an effect of beaver influence on DTW as a fixed effect (Appendix S2).

### Moose and elk populations

Several data sets, produced using different methods and spatial inference, but all with a nexus to the KV, are available to inform ungulate population trends and dietary composition. In 2003–2004, Dungan et al. (2010) estimated the number of resident moose for a 391‐km^2^ area west of the Continental Divide, including the KV valley bottom, through minimum count and individual identification of bull moose. An overall population estimate, including cows and calves, was derived by using bull‐to‐cow and calf‐to‐cow ratios and applied to the number of bull moose observed. Standard deviations were then employed to estimate a summer resident population range (Dungan, 2007; Dungan et al., 2010). In the summers of 2019 and 2020, moose populations were estimated via thermal infrared (TIR)‐assisted fixed‐wing aerial surveys. Sample transects represented a large portion of RMNP (694.7 km^2^), both east and west of the Continental Divide, including the KV (Figure 2). The TIR surveys supported the double‐observer (mark–recapture) distance sampling design we used with Bayesian hierarchal models to estimate the size of counted groups and moose abundance (Abouelezz & Hobbs, 2025). Transects were flown across 2 strata, informed by vegetation type, slope, and distance to surface water information. Strata were binned by moose habitat quality (high or low). Population estimates were developed for each stratum and for the entire study area.

The KV is primarily elk summer range; 75–90% of elk that use RMNP summer in the KV and nearby alpine tundra (Monello et al., 2006; NPS, 2007; Oldham, 2010). Many of these animals winter east of the Continental Divide, whereas the remainder move west of the park to lower elevations with thinner snowpack. Elk survey data in RMNP are available for the east side winter range, as described in the RMNP elk and vegetation management plan (NPS, 2008, 2007) but not the KV summer range. Animals that summer in the KV are captured in several different winter survey efforts east and west of the Continental Divide. RMNP makes up 18% of Colorado Parks and Wildlife's (CPW) Data Analysis Unit (DAU) E‐8, which covers approximately 2143 km^2^ west of the Continental Divide and includes the KV. The area of RMNP east of the divide is in CPW's DAU E‐9 and includes the park's elk winter range. The CPW reports modeled elk population size and observed demographic information annually for the E‐8 troublesome elk herd (1991–2023) and for the E‐9 Saint Vrain herd (1988–2023, with some data gaps). The model is informed by winter aerial survey data (Colorado Parks & Wildlife, 2024a, 2024b; Huwer, 2007; Oldham, 2010). Finally, Hobbs and Abouelezz (2020) estimated east side elk winter range population size and demography with a Bayesian hierarchal model. The model assimilates 4 time series of data: aerial counts, ground counts, sex and age classifications, and annual harvest outside the park to fit a matrix population model with 3 sex and age classes.

Previous researchers analyzed moose and elk diets in RMNP. In 2003–2004, Dungan and Wright (2005) estimated the summer and early fall dietary composition of moose in RMNP west of the Continental Divide, including the KV. Visual observations of bite counts were classified by species and leaf number consumed per bite. Informed by these observations, simulated moose bites were clipped from plants, dried, and weighed to determine the dried weight per bite by species. Fecal pellets were collected and analyzed to inform the percent diet composition by species. Information on the botanical and nutritional composition of elk summer diets (Baker & Hobbs, 1982) in RMNP was produced by observing elk bites by species over multiday sampling periods, and samples were collected for laboratory analysis following the observed foraging period.

### Willow

Maximum heights of willow plants were measured with a stadia rod in 3 exclosed and similar unexclosed areas outside each exclosure from 1997 to 2021. Exclosures were built to exclude moose and elk. Deer were present in low numbers during our POR and were not suspected to contribute to substantial willow browse. We analyzed willow species that can reach at least 3‐m tall (*Salix drummondiana* Barratt, *Salix geyeriana* Andersson, *Salix lasiandra* Bentham ssp. *lasiandra*, *Salix monticola* Bebb, *Salix bebbiana* Sargent. and *Salix planifolia* Pursh). Clonal forms of *S. planifolia* and *S. wolfii* Bebb were not included because plants are naturally <1.0‐m tall. The Colorado River trailhead and Gaskill exclosures (each 0.2 ha in size) were built in 1996, and willow height was measured along 30‐m‐long randomly located transects inside and outside exclosures. The Holzwarth exclosure (6.57 ha) was built in 2011, and willow height was measured along 5.66‐m‐long transects in randomly located plots inside and outside exclosures (Zeigenfuss et al., 2011). Annual sample sizes ranged from 60 to 474 willows inside and from 208 to 699 outside exclosures. Nonlinear trend in willow stem height over the POR was modeled using GAMs with exclosure as a factorial term (Appendix S2).

Willow polygons were mapped as tall (≥2.0 m) or short (<2.0 m) types by digitizing patch perimeters at a reference scale of 1:1000 in GIS with 1999 and 2019 georeferenced imagery. Patches of tall and short willows, as well as nonwillow areas of grassland or meadows, were visually distinguished based on texture and shadows cast by willow canopies (Giuliani et al., 2000). The 1999 imagery was 1:3600 scale USGS air photos for the KV inside RMNP and 1:24,000 scale Digital Orthophoto Quadrangles for the KV outside RMNP (EarthExplorer.USGS.gov). The 2019 imagery was from the National Agriculture Imagery Program (NAIP, 2021) at a 0.60‐m resolution. Willow height class polygons mapped from the 2019 imagery were calibrated using hand‐sampled plant height data from the RMNP Elk and Vegetation Management Monitoring Program (255 plants in 9 plots; NPS, 2007), Rocky Mountain Inventory and Monitoring plots (1571 plants in 27 plots [Schweiger et al., 2015]), and Contento (2021) (936 plants in 45 plots). Approximately 15% of the mapped 2019 polygons were field validated in 2021. No height data were available for calibration or validation of the 1999 map. Differences in median tall and short willow patch size and total area inside and outside RMNP were assessed with nonparametric Kruskal–Wallis and permutation tests (Appendix S2).

### Vegetation composition

Vegetation composition was measured in a 2‐m‐radius plot around each of 31 monitoring wells in 1998 and 2021. All plants within each plot were identified to species, and the percent canopy cover was visually estimated by the same 2 observers in both periods. Metrics summarizing invasive nonnative cover, conservatism (Wilhelm & Ladd, 1988), and wetland affinity were estimated for all plots and years based on species characteristics modified from Smith et al. (2020). Cluster, indicator species, nonmetric multidimensional metric scaling analyses of similarities, and distance‐based permutational multivariate analyses of variance were used to characterize changes in community composition (McCune et al., 2002; Oskanen et al., 2022) (Appendix S2).

## RESULTS

### Climate and WB

All models of climate and WB variables allowed a linear estimate of the POR trend. Models were improved by and had significant nonlinear within year (seasonal) and random annual terms, but because GAMs were additive, the overall POR trends were meaningful. No model had improved AIC scores when PDO was included, so we eliminated this variable. Most models explained a sizeable proportion of the total variance (Appendix S3).

The median GS air temperature at the Grand Lake COOP station from 1950 to 2023 increased by 2.3°C from a first‐decade average of 10.1 to 12.4°C in the last decade (*p* < 0.0001) (Figure 3a). Total summer precipitation decreased 54.4% to 25.7 mm (*p* = 0.015) (Figure 3b). April 1 maximum SWE at Lake Irene decreased 16.2% to 116 mm, but this trend was only marginally significant (*p* = 0.069) (Figure 3c). Several WB variables changed from 1980 to 2023. Total monthly climatic deficit increased by 14.4% to 3.1 mm (*p* = 0.038) (Figure 3d), and total PET increased by 11.1% to 5.1 mm (*p* < 0.0001) (Figure 3f). Cumulative runoff exhibited no temporal change (*p* = 0.51) (Figure 3e)

FIGURE 3(a) Median growing season (GS) monthly air temperature, (b) total GS monthly precipitation (mm) at the Grand Lake 1 northwest station 053496, (c) maximum annual week of 1 April snow water equivalency (SWE) at the Lake Irene SNOWTEL station 565, (d) total GS monthly climatic deficit, (e) total GS monthly cumulative runoff, (f) total GS monthly potential evapotranspiration, (g) mean GS monthly Colorado River flow at U.S. Geological Survey gage 09010500, (h) flow variability as the standard deviation of logged GS monthly flow, and (i) proportion of flow in the Colorado River versus the Grand Ditch (at State of Colorado Division of Water Resources gage GRNDRDCO). In (d) and (g), relation shown is for a central location in the Kawuneeche Valley from 1980 to 2023. Fitted lines and confidence intervals (dashed lines) are for the period of record (POR) term in each trend model (i.e., they are not direct fits to the points in each panel). All significant *p* values for the POR trends are <0.05 with the exception of SWE (*p* = 0.069). Additional trend model details are in Appendix S2.

**Figure:**
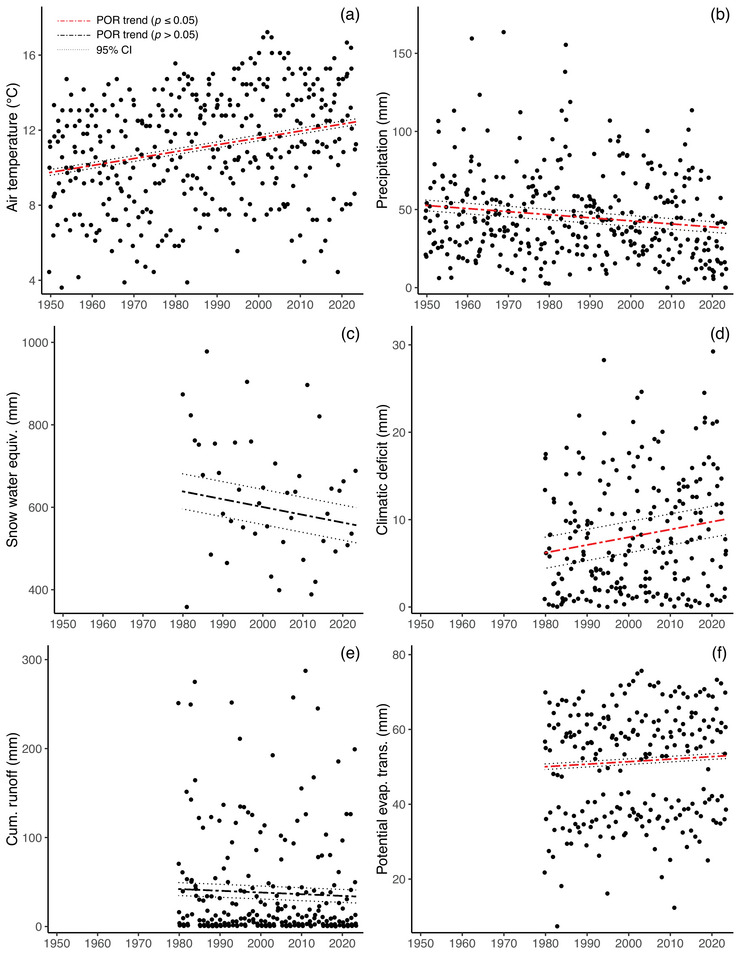

**Figure:**
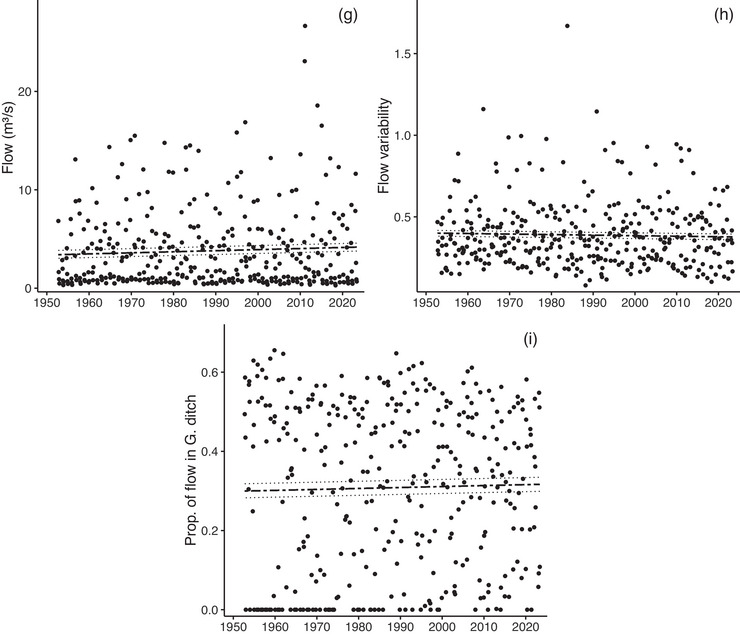

### Surface water

GS Colorado River discharge averaged 2.69 m^3^/s from 1953 to 2023, and there was no meaningful trend over the period (*p* = 0.28) (Figure 3g). Variation in discharge also did not change over time (mean standard deviation of log[flow] of 0.38) (*p* = 0.58) (Figure 3h). Likewise, the fraction of Colorado River flow diverted by the Grand Ditch did not change over time (mean of 0.315) (*p* = 0.58) (Figure 3i). Results from models of hydrologic responses showed significant within‐ and across‐year effects and no clear role of a PDO (Appendix S4).

Total area of ponded water in the KV declined by 94% from 1953 (71.4 ha) to 2019 (4.04 ha) (Appendix S4). Mean pond size declined nonlinearly, from 0.10 to 0.04 ha over this period (Appendix S4), with the most rapid change occurring after 1999, suggesting that losses in area were uniformly distributed across pond size from 1953 to 1999 but that more recent losses were likely due to a reduction in the area of larger ponds.

### Groundwater

**FIGURE 4:**
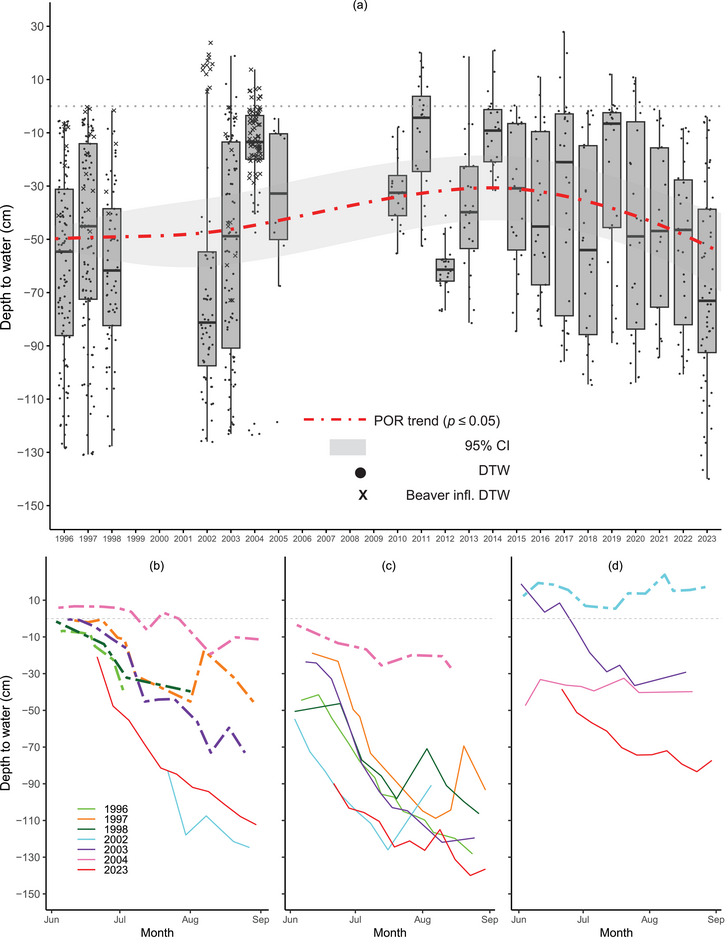
Growing season depth to groundwater (DTW) at select sites in the Kawuneeche Valley, Rocky Mountain National Park, from 1996 to 2023: (a) weekly DTW across 10 comparable wells grouped by year (points, observations not influenced by beaver dams; crosses, observations influenced by beaver dams; red dashed line, nonlinear trend at *p* < 0.05; whiskers, 95% confidence intervals around growing season [GS] weekly DTW) and (b–d) DTW across GSs at 3 individual wells (heavy dashed lines, GS with direct beaver influence). Trend model details are in Appendix S2.

Overall, DTW changed little from an average of approximately −50 cm in 1996 to −55 cm in 2023. The trend was nonlinear across the POR with a period of shallower water tables in the early 2010s, followed by a decline to a deeper water table through 2023 (Figure 4a; Appendix S5). The shallower water tables corresponded to higher GS flow in the early 2010s in the Colorado River (Figure 3g; Appendix S5). However, years with nearby beaver dams on the Colorado River (1996–1998, 2002, and 2004) had markedly shallower DTW than years without beaver dams (*p* < 0.0001) (Figure 4b–d). This beaver effect persisted across each GS when a dam was present and created the shallowest water tables in our data sets. There were strong site effects indicating that trends in groundwater levels were spatially heterogeneous (*p* < 0.0001). The DTW response to Colorado River flow was best modeled as nonlinear, with increased flow leading to higher groundwater up to a threshold of around 15.8 m^3^/s above which there was little change in DTW. Our groundwater data suggested autocorrelation among wells, and these results should be used with some caution.

### Moose and elk

The resident summer moose population west of the Continental Divide (391 km^2^) in RMNP, including the KV valley bottom, was estimated to be 54–59 and 37–43 animals in 2003 and 2004. Based on a low of 37 animals and a high of 59 animals, we derived an estimated density of 0.095–0.151 moose/km^2^ (Dungan, 2007; Dungan et al., 2010). Abouelezz and Hobbs (2025) produced summer population estimates of 149 animals (95% highest density interval [HDI] 100, 198) in the larger study area (694.7 km^2^) in 2019 and 143 (HDI 97, 189) in 2020. Mean moose density for the study area was estimated at 0.215 moose/km^2^ (HDI 0.145, 0.286) in 2019 and 0.207 moose/km^2^ (HDI 0.144, 0.276) in 2020. Mean moose densities in the higher moose habitat suitability transect stratum (flatter slope, riparian vegetation, near‐surface water), including the KV, were 0.39/km^2^ (HDI 0.26, 0.523) in 2019 and 0.28/km^2^ (HDI 0.182, 0.382) in 2020.

Elk population estimates in CPW's DAU E‐8 decreased 31.7% from a high of 5901 animals in 1995 to the most recent estimate of 4029 animals in 2023 (Colorado Parks & Wildlife, 2024a) (Appendix S6). Similarly, DAU E‐9 decreased by 51.6% from a high of approximately 4400 animals in 1999 to 2130 animals in 2023 (Colorado Parks & Wildlife, 2024b; Huwer, 2007). The RMNP east side winter range estimates peaked at ∼1500 animals in the early 2000s and declined steadily to a median estimate of 124 animals (HDI 62, 195) during the winter of 2018–2019 (Hobbs & Abouelezz, 2020).

### Willow

**FIGURE 5:**
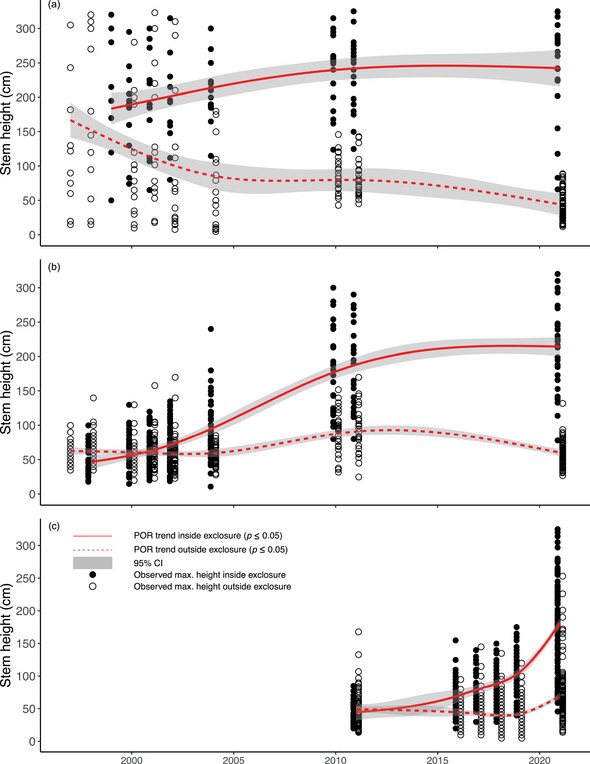
Maximum willow stem height inside and outside elk and moose exclosures in the Kawuneeche Valley, Rocky Mountain National Park, from 1997 to 2021: (a) Colorado River trailhead, (b) Gaskill, and (c) Holzwarth. Sample sizes of measured stems vary by year and exclosure. Trend model details are in Appendix S2.

Median willow heights outside the Colorado River trailhead exclosure declined 78% from 171 cm in 1997 to 38 cm in 2021, whereas inside the exclosure height increased 52% from 205 to 311 cm during the same period (Figure 5a). Trajectories of height change diverged inside versus outside the exclosure just 2 years after its construction (*p* < 0.0001 for all exclosures) (Appendix S7). In 1997, when the Gaskill exclosure was constructed, willows were already heavily browsed with a median initial willow height of 60 cm (Figure 5b). Elimination of browsing inside the Gaskill exclosure resulted in a 498% height increase to a mean of 239 cm in 2021. Willows inside the Holzwarth exclosure, built in 2011, increased 338% from a median height of 45.5 to 199 cm in 2021, whereas outside the exclosure median height increased slightly from 40.5 to 60 cm (Figure 5c). Similar trends in plant height inside and outside exclosures are likely due to the slow recovery of some formerly browsed plants that lack beaver‐influenced water tables, periodic moose entry by jumping over exclosure fences to eat willows, and shoots that are protected by dead branches outside exclosures.

From 1999 to 2019, the total area and median patch size of tall willows in RMNP declined, whereas those of short willows increased (Appendix S7). Willows ≥2.0‐m tall covered 283 ha in 1999 and declined by 98% to 7 ha in 2019 (*p* < 0.0001), whereas the area of willows <2.0‐m tall increased by 204% from 116 to 353 ha (*p* = 0.01). Tall willow median patch size declined from 0.42 ha in 1999 to 0.08 ha in 2019 (*p* < 0.0001), whereas short willow median patch size did not change (*p* = 0.6).

### Vegetation composition

**FIGURE 6:**
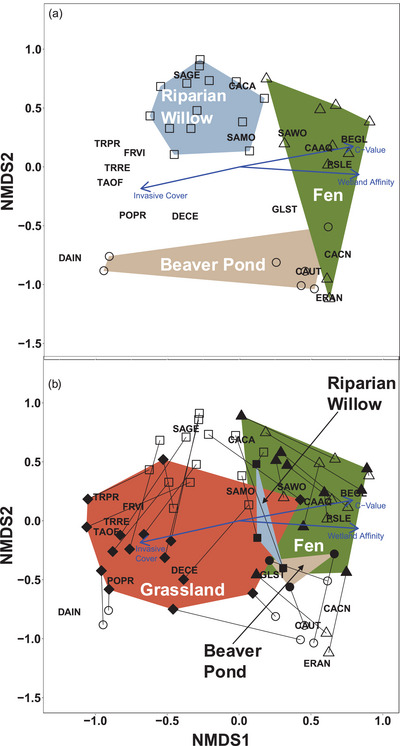
Ordination of vegetation at 31 sites in the Kawuneeche Valley, Rocky Mountain National Park, in (a) 1998 for fens (triangles), beaver ponds (circles), and tall willow riparian areas (squares) and (b) 2021 for fens, beaver ponds, tall willow riparian areas, and a novel grassland type (diamonds) (symbols, sample sites; open symbols, 1998; solid symbols, 2021; indicator species: BEGL, *Betula glandulosa*; CACA, *Calamagrostis canadensis*; CAAQ, *Carex aquatilis*; CACN, *Carex canescens*; CAUT, *Carex utriculata*; DAIN, *Danthonia intermedia*; DECE, *Deschampsia cespitosa*; ERAN, *Eriophorum angustifolium*; FRVI, *Fragaria virginiana*; GLST, *Glyceria striata*; PEFL, *Pentaphylloides floribunda*; POPR, *Poa pratensis*; PSLE, *Psychrophila leptosepala*; SAGE, *Salix geyeriana*; SAMO, *Salix monticola*; SAWO, *Salix wolfii*; TAOF, *Taraxacum officinale*; TRPR, *Tragopogon pratensis*; TRRE, *Trifolium repens*). Both years appear in (b) (thin black lines, direction and magnitude of the shift in each site across the 2 periods; blue arrows, year‐specific relationships with 3 vegetation metrics). Ordination model and vegetation metric details are in Appendix S2.

Vegetation composition was distinct among wetland types in 1998 and 2021 (*p* = 0.0001 for both years) (Appendix S7). In 1998, 3 distinct types occurred: riparian tall willows, beaver ponds, and fens (Figure 6a). Fens dominated by *Carex aquatilis*, *Betula glandulosa*, and *S. planifolia* were present along valley margins where hillslope groundwater inputs support shallow and persistent groundwater. Riparian wetlands occupied the valley bottom on alluvium and were dominated by *S. geyeriana* and *S. monticola* with an understory of *Calamagrostis canadensis*. Beaver ponds were common in 1998 and dominated by *Carex utriculata*.

By 2021, a fourth novel elk–moose–grassland community type had developed (Figure 6b; Appendix S7) from formerly tall willow and beaver pond sites. The novel community was marked by a strong association with non‐native invasive plant species cover (1998 *p* = 0.004; 2021 *p* = 0.001), including *Cirsium arvense*, *Phalaris arundinacea*, *Poa pratensis*, *Taraxacum officinale*, and *Trifolium repens*. The riparian willow, fen, and beaver pond communities that did not undergo a state transition were similar in composition to 1998 but had less willow (mean of 17.8% in 1998 to 1.8% in 2021). *Calamagrostis canadensis* cover in fens increased from 18.0% to 36.3%, and *Deschampsia cespitosa* cover decreased from 19.7% to 3.8%. The remaining willow and fen sites retained species with higher conservatism (1998 *p* = 0.001; 2021 *p* = 0.001) including *Polemonium occidentale*, *Cardamine cordifolia*, and *Pedicularis groenlandica*. These sites also had higher wetland affinity (1998 *p* = 0.001; 2021 *p* = 0.001) with several obligate species including the 3 tall willows and *Carex utriculata*.

## DISCUSSION

### Characterization of the initial state

The KV environmental history documents the initial beaver–willow state with abundant tall riparian willow stands and persistent and widespread beaver activity. Historic ground photographs from 1889 to 1922 help visualize this state (Appendix S8). Extensive written history by early settlers also documented beaver complexes supported by tall willow stands in the 19th and early 20th centuries (Andrews, 2015), as has been reported across the Rocky Mountains (Levine & Meyer, 2019). Willow pollen fluxes have been relatively constant for more than 9000 years (Fall, 1997), indicating the potential long‐term presence of the beaver–willow state. Tall riparian willow communities occurred in a complex matrix with beaver dams and ponds that flooded large areas and created vital disturbances and soil moisture conditions for willow establishment and growth. Groundwater‐supported fens with peat soil have been present for thousands of years, along with marshes on beaver pond margins (Chimner & Cooper, 2003a). Understanding the KV environmental history is critical to interpreting the drivers of ecological collapse and the factors that make the new state stable. It also is critical for defining risks for ecosystem collapse elsewhere in RMNP and in other protected areas and for evaluating options for ecosystem recovery (Bland et al., 2018).

### Factors leading to ecosystem collapse

Human influences on KV ecosystems began well before the 1915 establishment of RMNP (Schweiger et al., 2016). Native Americans were skilled hunters and lived in the KV for millennia (Andrews, 2015). Along with humans, the apex predators grizzly bears (*Ursus arctos*) and wolves (*Canis lupus*) would have kept large ungulates, other mammals, and vegetation in dynamic equilibrium. Fur trappers nearly eradicated beaver in the early to mid‐1800s, effectively interrupting for many decades the beaver's Holocene influence on river dynamics and floodplain formation (Westbrook et al., 2006, 2011). Nearly all apex predators were extirpated in the late 19th and early 20th centuries (Laliberte & Ripple, 2004). Homesteaders and miners arrived in the late 1800s and removed riparian willows from several areas to create pastures and ditches for drainage and irrigation, removed beaver, and introduced plants native to central Europe for hay production. The Grand River Ditch was constructed from 1890 to 1936 and has reduced summer Colorado River flows ever since. Native elk were nearly extirpated by market hunting and then reintroduced from a herd in Yellowstone National Park in 1913–1914. Hunting outside the park and occasional culling of elk on the eastern side winter range of RMNP moderated browsing pressure, despite the near absence of apex predators (Andrews, 2015). These events altered KV riparian ecosystems but did not trigger its collapse because beaver recolonized and increased to a population estimated to be in the hundreds by 1940 (Packard, 1947) and because tall willow were abundant until the late 1990s.

Tall riparian willow communities covered much of the KV throughout the 19th and 20th centuries (Andrews, 2015). Willow height outside exclosures declined across our POR, and by 2021, tall willow in the RMNP portion of the KV occurred primarily within ungulate exclosures. Tall willow species in RMNP are critically linked by their life‐history traits to the ecological and hydrological processes of riparian areas, but herbivory can disrupt reproduction. Willow seeds are produced in catkins that form on the previous year's stem growth. Most tall willow species flower in early spring and their small aerially dispersed seeds must land on bare and wet mineral soil to germinate. Bare and wet sediment occurs on active point bars created by fluvial processes (Woods & Cooper, 2005), as well as on beaver‐created pond margins and abandoned stream channels (Cooper et al., 2006a). Seed rain in heavily browsed environments may be very low (Bilyeu et al., 2008; Gage & Cooper, 2004) because the previous year's stem growth is the most desirable browse for ungulates, limiting seed production and seedling establishment (Wolf et al., 2007). Excessive herbivory and alteration of food webs by moose and elk can have dramatic effects (Hobbs et al., 2024; McLaren et al., 2004). Following the reintroduction of elk to RMNP in the early 20th century, their numbers increased, and by 1940, competition between elk and beaver was noted (Packard, 1947). Elk population control was utilized to maintain the RMNP population near 500 animals until 1968 when a so‐called natural regulation policy was adopted. Following the implementation of this policy, the winter range population in the east side of RMNP rose to ∼1500 animals by the early 2000s (Hobbs & Abouelezz, 2020; Lubow et al., 2002), but it has declined since then because many elk migrate to lower elevations in winter, as they likely did historically (Hobbs & Abouelezz, 2020). Although RMNP‐specific elk data for the KV are not available, population estimates for CPW's E‐8 troublesome herd that includes the KV indicate that regional elk numbers were highest during the mid‐1990s to early 2000s. However, by 2023, the E‐8 elk population had declined almost 32% from its peak, and the E‐9 elk population had declined over 51%. The RMNP‐specific elk population size estimates on the east side winter range showed even sharper declines. Although these data series did not perfectly describe the KV, they did connect to the KV because many RMNP winter range elk (a subset of E‐9 animals) and a subset of E‐8 elk summer in the KV (Monello et al., 2006; Oldham, 2010).

Moose arrived in the KV in 1980 (Stevens, 1988) after introduction outside RMNP by the State of Colorado. By 2003–2004, the KV summer range resident moose density was calculated to be 0.095–0.151 moose/km^2^ (Dungan, 2007). Based on the energy requirements and digestible energy intake of a 344‐kg male moose, the carrying capacity during this period was estimated to be 0.21 moose/km^2^ (Dungan et al., 2010). More recently, Abouelezz and Hobbs (2025) estimated mean summer moose densities across their study area at 0.215 moose/km^2^ (2019) and 0.207 moose/km^2^ (2020). This suggests that moose density in the larger landscape that included the KV may have increased by as little as 37% and as much as 126%. In 2019 and 2020, the mean summer moose density in areas with more moose habitat, which largely included the KV valley bottom, was 0.39 moose/km^2^ (2019) and 0.28 moose/km^2^ (2020) (Abouelezz & Hobbs, 2025). We did not have an estimate of moose carrying capacity for the KV circa 2020. However, willow biomass was much greater in the early 2000s than in the early 2020s, suggesting that any previously estimated KV nutritionally based carrying capacity would be dramatically exceeded by the estimated moose densities in the KV today.

Up to ∼90% of moose summer diet in the KV is willow (Dungan et al. 2010), with adult male moose consuming up to 10 kg of dry matter per midsummer day. Over a 3‐ to 4‐month GS, one animal may consume a ton or more of dry plant matter (Dungan & Wright, 2005). In contrast, elk summer diets are largely composed of grasses and sedges, with shrubs and dicots used in lesser amounts (Baker & Hobbs, 1982). Shrub consumption by elk did not exceed 22% of their summer diet, and willow species consumption did not exceed 16%. Although willows outside exclosures are very short, moose and, to a lesser degree, elk continue to browse these plants. Although the methods and inference areas for elk and moose population estimates differed through time, data on ungulate density suggest that elk likely declined in the KV since the mid‐1990s to early 2000s, moose density increased, and willows steadily declined. Although there is no ungulate browse information specific to the KV, the density, seasonality, and diet differences of moose and elk suggest that moose are likely to have contributed more substantially to willow decline. Further research is needed to better understand elk and moose population information in the KV to best guide site‐specific management.

After being nearly extirpated by early 19th‐century fur trappers, beavers likely reestablished in the KV by the 20th century. The legacy effects of beaver's landscape engineering (Albertson et al., 2022) allowed them to reoccupy relict dam and pond complexes and build new dams. In the 1930s and 1940s, Packard (1947) estimated that hundreds of beavers were in the KV. A beaver family requires approximately 4 ha of tall willows with a density of at least 10 stems·m^−2^ to persist in RMNP (Peinetti et al., 2009). Where heavy browsing of tall willow stems occurs, it typically precedes the decline of beaver populations, and willow stand condition can predict potential beaver populations (Baker et al., 2012). When beavers are absent for many decades, streams can incise and no longer provide the substrate necessary for willow establishment, even when stream flows do not change, such as in the KV. The loss of beaver dams reduces river–riparian hydrologic connectivity limiting opportunities for willow establishment, and the Colorado River now only floods its immediate streamside (Westbrook et al., 2006).

The 54% reduction in summer precipitation and 11% increase in evapotranspiration in the KV likely exacerbated the water table decline when beaver dams were absent. Willow can adapt to drier climate conditions by becoming more water‐use conservative (Butterfield et al., 2021). However, the added pressure of persistent ungulate browsing on willow, along with sapsucker (*Sphyrapicus nuchalis* and *Sphyrapicus varius*) feeding activity that opens wounds in tall willow stems, inadvertently allowing the fungus *Cytospora chrysosperma* to infect and kill stems (Kaczynski et al., 2014), exacerbated tall willow decline and contributed to unsuitable conditions for beaver occupancy. Following their recovery from the fur trapping that initially decimated their populations, beavers were once again no longer present in the KV after 2004, this time due to habitat loss. Recently, beaver activity has been observed in one KV ungulate exclosure.

**FIGURE 7:**
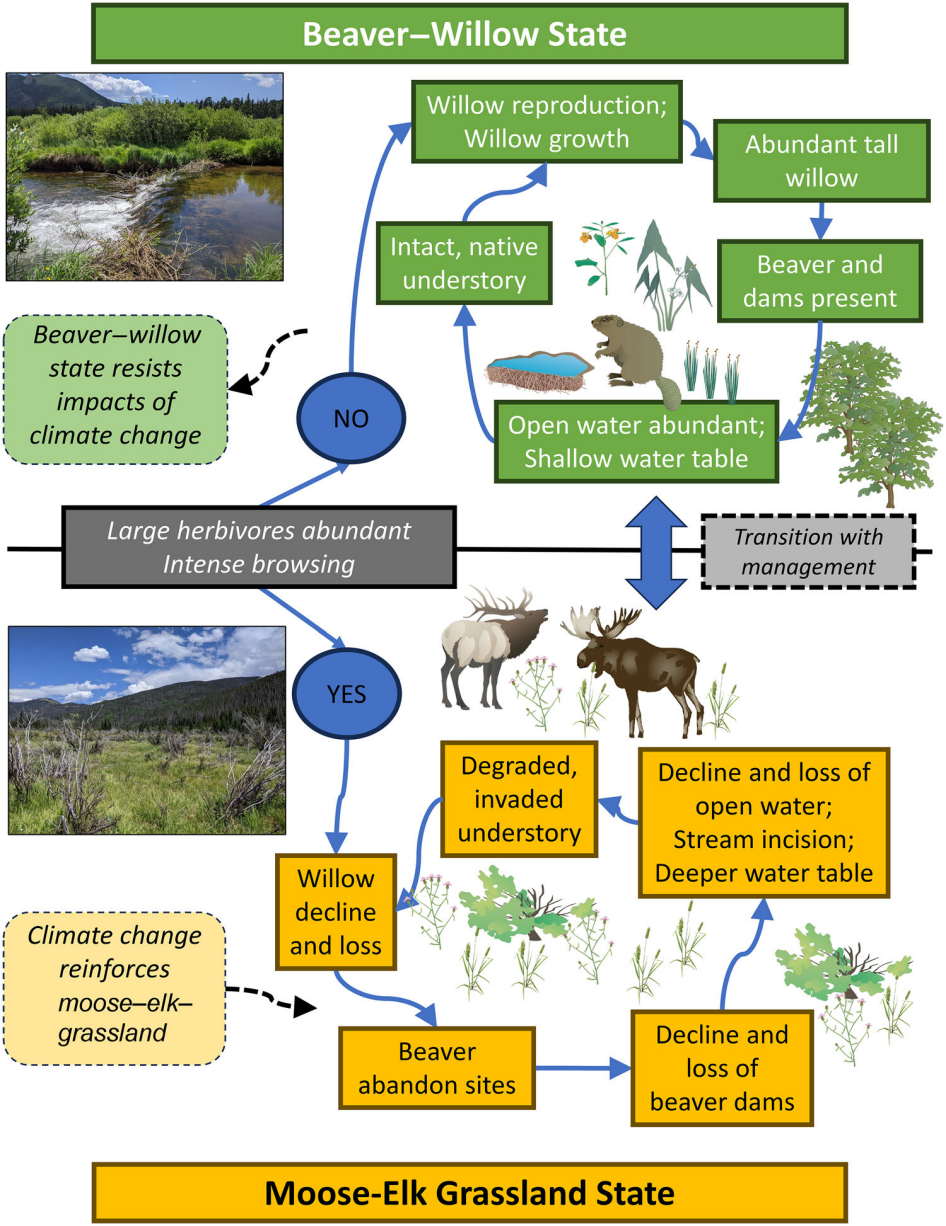
Conceptual diagram of key interrelationships driving wetland processes in the Kawuneeche Valley, Rocky Mountain National Park (dark gray box, central dichotomy; green, beaver–willow state; yellow, moose–elk–grassland state; top inset, an active beaver dam and relatively tall willow typical of the beaver–willow state; bottom inset, dry grassland with heavily browsed and damaged willow and invasive plants common in the moose–elk–grassland state). Animal and vegetation graphics are courtesy of Integration and Application Network, University of Maryland Center for Environmental Science (ian.umces.edu/symbols/).

Figure 7 shows a conceptual model of the key ecological processes in the beaver–willow state collapse in the KV. The original beaver–willow state featured abundant tall willow stands, beavers and beaver dams, large areas of open water and locally high‐water tables, native understory species, and reproduction of willows. This state was buffered from climate changes with its relatively abundant surface and groundwater, shade‐forming canopies, and ubiquitous stream‐floodplain interactions. Large herbivores were present but their effects were moderated by apex predators prior to the 20th century, low ungulate populations until the middle to late 20th century, and the beaver–willow state's resistance to change. However, increases in large herbivore browsing (up to 126% increase in moose density) during the first 2 decades of the 21st century and *Cytospora*‐influenced stem mortality likely played a pivotal role in the rapid decline of the tall willow canopy. Without tall willows, beavers had neither the food nor building material to persist and abandoned the area in the early 2000s. With the erosion of beaver dams, open water was lost, water tables near the ponds declined, and non‐native plants invaded the newly dried areas. In this new moose–elk–grassland state, the effects of climate change reinforce the state change. Positive feedback was created by the loss of open water, lower willow productivity, climatic‐driven changes in WB, and beaver absence that prohibited the natural reestablishment of the beaver–willow state. A similar elk–grassland state developed by the 1950s in Yellowstone National Park and persists today (Hobbs et al., 2024; Wolf et al., 2007). It is likely that the elimination of the potential for beaver occupancy is a principal mechanism behind the hysteresis in both of these iconic protected areas and that it perpetuates the new alternative stable state.

### Best management approaches

Management options for the KV are complex and likely limited to resisting ecological transformation through proactive restoration of the beaver–willow state; directing change through vegetation management that likely would still result in a grassland ecosystem, but one with more native and fewer invasive, non‐native species; or accepting change to the moose–elk–grassland state through a no‐action approach (Schuurman et al., 2022). Restoration of the beaver–willow state would reinitiate lost ecosystem services, including high biodiversity provisioning and enhanced capacity to resist or buffer floods, droughts, and wildfires, which are anticipated to become more frequent and severe as the climate continues to change (Jordan & Fairfax, 2022; National Park Service, 2024). Although hysteresis is preventing or restricting return to the original beaver–willow state, it appears that a short window of opportunity may exist to employ management actions to disrupt the hysteresis and set a trajectory for restoration of the KV. We found little evidence of a change in the Colorado River flow in the KV, and SWE in the watershed only marginally declined in the period of riparian ecosystem collapse. Likewise, groundwater depths did decline near abandoned beaver ponds, but not in all areas of the valley. The snowmelt‐dominated hydrologic regime of the KV is a key stabilizing factor of the climate support system, as seen at larger scales (Feng & Gleason, 2024; Harder et al., 2015).

Potential management actions for recovery of the beaver–willow state are not straightforward. Conflicts have arisen in national parks, like RMNP, where protection may contribute to the unintended consequences of concentrating large ungulates, including newly arrived moose, in areas where hunting is prohibited. With the near absence of apex predators, the effects of ungulate browsing can lead to degradation and challenge the NPS's ability to achieve its core mission (Hobbs et al., 2024). The moose–elk–grassland state developed where willow height was dramatically reduced and willow establishment ceased because hydrologic connections were minimized due to beaver absence. However, within fenced exclosures, willow height has rapidly increased, suggesting that the climate and hydrologic conditions are still suitable for tall willow communities in the early 2020s. It took 2–5 years for newly exclosed willows in the KV to grow significantly taller than browsed willows and 10–20 years for them to grow large enough to potentially support beaver. Beaver activity establishes natural hydrologic connectivity between streams and their floodplains, and willow establishment is largely dependent on beaver activity. Most remaining willows in the study area are many decades old and declining; there has been little recent establishment. The death of existing willows could necessitate planting willows in future restoration projects, an expensive activity that would be successful only in areas with appropriate groundwater depths.

Our results indicate that for long‐term restoration to be successful, herbivory must be reduced. However, RMNP and many other protected areas have limited options for reducing herbivory. Physical barriers in parks and state‐managed hunter harvests on land surrounding parks are important options. Ungulate exclusion fencing can reduce browsing pressure but is only feasible in limited areas. Ungulate population reduction is an option that could allow larger scale reduction in herbivory; however, because willow biomass is reduced, it takes fewer and fewer ungulates to remove their annual biomass. In addition, trade‐offs exist as moose and elk population reduction could limit wildlife viewing opportunities for the public. The effect of the reintroduction of the apex predator gray wolves on riparian ecosystem recovery is uncertain. Gray wolves were reintroduced to Yellowstone National Park in 1995–1996 (Smith et al., 2020), but their role in ecosystem recovery has been limited (Brice et al., 2024; Hobbs et al., 2024). Wolves were reintroduced to Colorado in December 2023 under State Statute 33‐2‐105.8 and are already in the vicinity of RMNP, but their effect will likely be limited because of the small size of RMNP, complex sociopolitical dynamics surrounding the reintroduction program, and the limited effects of wolf predation on ungulates as it relates to riparian system recovery in the Rocky Mountains. Achieving a sustainable population of elk is a key management objective of the RMNP elk and vegetation management plan (NPS, 2008, 2007) and includes the recovery of tall willows, beaver, and other native biota. A management goal for moose has not been established.

Also needed for long‐term restoration to be successful is the reestablishment of river and riparian hydrogeomorphic connectivity. This will supply fresh sediment for willow seedling establishment and raise the water table to sustain willow growth. Even with a reduction in ungulate herbivory, the current conditions in the KV are not suitable for widespread beaver occupancy. To improve conditions for beaver, many areas in the western United States have used biomimicry, such as beaver dam analogs (Bouwes et al., 2016), to raise stream stage and, in some instances, to raise local water tables of degraded riparian areas to increase rates of willow regrowth (Hobbs et al., 2024; Marshall et al., 2013). Although ungulate management may be effective at a large spatial scale, ungulate exclusion, water table enhancement, and vegetation management are tools that could enable managers to begin restoration in the most critical areas. Given current and projected future climate changes in RMNP (National Park Service, 2024) and the likely impacts of climate change on wetland ecosystems (Carroll et al., 2024; Hogan & Lundquist, 2024), restoring the willow–beaver state may be time sensitive. Future studies could guide understanding of the relative effects of hydrologic processes, climate, and herbivory in willow growth and reproduction.

Protected areas, such as RMNP, are not immune from ecosystem collapse and the development of alternative stable states (Bergstrom et al., 2021). Their protected status may indirectly contribute to it. A hands‐off management approach in the absence of human and wildlife predators has created unintended outcomes. The strength of hysteresis, due to continued heavy browsing and potential climate changes, indicates that natural conditions for return of the beaver–willow state do not exist. The decline of native species and ecosystem function in KV riparian areas cannot recover within multidecadal time scales unless they are aided by human actions.

## AUTHOR CONTRIBUTIONS

The concepts in this paper were developed by all the authors. The manuscript draft was written primarily by D.C. with contributions by W.S., C.W., and H.A. All members provided editing and contributed to the final document. Data collection was by all authors, and analyses were led by W.S. with contributions from all other authors.

## Supporting information



Supporting Information
