# Supplementary material for: Rapid riparian ecosystem decline in Rocky Mountain National Park
Source: Conserv Biol. 2025 May 31;39(5):e70053. doi: 10.1111/cobi.70053 (PMC12451483; doi:10.1111/cobi.70053)
Supplement: Supplementary file 1 — Supporting Information [file COBI-39-e70053-s001.pdf]

Appendices

Rapid decline in riparian ecosystems in Rocky Mountain National Park

David J. Cooper[1], E. William Schweiger[2], Jeremy R. Shaw[1], Cherie J. Westbrook[3], Kristen Kaczynski[4], Hanem Abouelezz[5], Scott Esser[5], Koren Nydick[5], Isabel de Silva[6], Rodney A. Chimner[7]

[1]Department of Forest and Rangeland Stewardship, Colorado State University, Fort Collins, CO 80523, U.S.A.

[2] National Park Service, Rocky Mountain Inventory and Monitoring Network, Fort Collins, CO, 80525, U.S.A.

[3] Department of Geography and Planning and Centre for Hydrology, University of Saskatchewan, Saskatoon, Saskatchewan, Canada S7N 5C8

[4] Department of Earth and Environmental Sciences, California State University, Chico, CA, 95929, U.S.A.

[5] National Park Service, Rocky Mountain National Park, Estes Park, CO, 80517, U.S.A. (currently National Park Service, Natural Resource Stewardship and Science Directorate, Fort Collins, CO, 80525, U.S.A.)

[6] National Park Service, Rocky Mountain National Park, Estes Park, CO, 80517, U.S.A.

[7] College of Forest Resources and Environmental Sciences, Michigan Technological University, Houghton, MI, 49931, U.S.A.

Corresponding Author

David J. Cooper, David.Cooper@colostate.edu, Department of Forest and Rangeland Stewardship, Colorado State University, Fort Collins, CO, 80523, U.S.A.

Appendix S1: Study area

The Kawuneeche Valley is bisected by the Colorado River channel that ranges from 5 to 10 m wide with a riparian zone 0.7 to 1.2 m above the river (Woods 2001), and up to several hundred meters wide (Figure 2 in main text). The valley is bordered on the east by the Front Range consisting of Precambrian metamorphic rocks and on the west by the Never Summer Range of upper Oligocene granitic magmas. Valley slopes are covered by Pleistocene era lateral moraines (Braddock and Cole 1990), with alluvial fans along hillslope margins. Organic soils 0.4 to >1.5 m thick are present in fens along valley margins in many areas supported by hillslope ground water, and silt loam. Loamy sand texture mineral soils elsewhere in the valley average 0.9 m thick. The valley is underlain by 3 to 4 m of gravel alluvium, with 15–122 m of Holocene and upper Pleistocene alluvium below that (Braddock and Cole, 1990).

Appendix S2: Methodological and analysis details

All analyses in this paper were conducted using R version 4.3.3 (R Core Team 2024) with libraries and functions as detailed below. All Geographic Information System (GIS) based work was done using ArcGIS Pro version 3.1 (ESRI 2023). Data and select R code are housed on Dryad (DOI pending).

Water balance

We estimated a daily water balance (WB; Lutz et al. 2010, Thoma et al. 2020) at a central location near vegetation and ground water sites (see Figure 2, main narrative for this location). Our WB model partitions precipitation (from DAYMET version 4; Thorton et al. 2021) into rain or snow following Jennings et al. (2018), the latter of which accumulates until temperatures (also from DAYMET) become warm enough to melt the snow (Hock 2003). We estimate a direct runoff parameter following NRCS (2017). We calculate potential and actual evapotranspiration following Oudin et al. (2005) with slope and aspect estimated from a digital elevation model to account for a localized heat load. We use SSURGO soil data for an initial soil water holding capacity (NRCS 2021). Subsequent soil water estimates account for actual evapotranspiration and an estimate of shade from plot level vegetation data. Climatic water deficit is estimated as the amount of additional water vegetation would use if available, calculated as the difference between actual and potential evapotranspiration (Stephenson, 1998). The model calculates water in excess of soil storage capacity as runoff. Runoff values are used to accumulate a daily cumulative runoff or "drainage" term following Croke et al. (2005) We assume a unit hydrograph to derive coefficients for "quick" or event-based runoff and "slow" or base runoff. These coefficients are optimized with calibration to DTW by iterating parameters singly and as pairs across a range of values, maximizing adjusted r^2 with DTW.

General additive models of climate, water balance, hydrology and willow stem height

We restrict most data in this paper to a May – September growing season under the assumption that this is the portion of the water year that is likely most important to vegetation and ungulate use of vegetation. A growing season-based time step also reduces variance within and across years. We summarize most variables as medians across each growing season. Medians are a preferred summary statistic for data with non-normal distributions, outliers and high variance. (i.e., Wilcox et al 2018). Most of our climate and hydrology data are of this form (Steinschneider et al. 2015). For variables where the response of interest is a sum (i.e., precipitation) we instead use a total across each growing season. We plot monthly (or weekly for ground water) point values in Figure 3 and 5 in the main narrative but expect readers to focus on the fitted lines in these Figures which reflect the overall period of record trends (slope, intercept, and point wise confidence intervals, see below).

We use Generalized Additive Models (GAM; Wood 2011, 2017) for most analyses. GAMs are well suited to data that are inherently nonnormal and that can have nonlinear relationships with their predictors. A GAM allows non-linear or "smooth" functions to be applied to all or a subset of predictor(s) as patterns in data indicate. Smooths are regularized nonparametric functions that can fit simple to very complex non-linear relationships. GAM models may also include predictors with a linear relationship with the response and/or with an *a priori* or defined non-linear relationships, such as a log. GAMs are additive, as the name suggests, allowing interpretation of each variable in the model independent of the others. Each smooth or linear term represents the unique effect of a single predictor on the response variable, controlling for the other predictors. The effect of each variable is assessed without interactions, meaning that the model assumes the effect of one predictor does not

change based on the value of another predictor. All GAM models were estimated using the mgcv package in R (Wood 2023).

Models were developed using a model selection process. We first estimated an intercept only null model. Variables were added and retained with four selection criteria: 1) change in Akaike Information Criterion (AIC; Venables and Ripley 2002) with a delta AIC threshold value of 2.0; 2) overall model and individual predictor interpretability; 3) adjusted r^2 values; and 4) a candidate predictor's p value. In general, we use a structural approach following Grace and Irvine (2020) and initially developed and included candidate predictors as those with likely direct or readily explainable indirect connections to a response.

GAMs with sufficient sample size within a growing season used a mixed model with years treated as a random factorial term and all other predictors fixed. All time-based terms in models were centered and normalized. Where exploratory work suggested a linear trend was present and also improved a GAMs quality, we allowed a linear or parametric POR trend estimate. In models that included a month within each growing season effect, these relationships were clearly non-linear and estimated as a smooth. The total number of observations for climate models was 365 (5 months across 73 years from 1950 to 2023) while water balance responses was 215 (data spanned 1980 to 2023) and for flow responses that began in 1953, the total number of observations was 350. Estimates of the period of record trend had effective sample sizes set by the number of years in the model (i.e., 73).

The GAM of DTW included sites as a random term and was improved by additional predictors for Colorado River flow, one year lagged snow water equivalent (SWE; both best fit as a smooth) and a linear term for climatic deficit. The lagged SWE term likely captures lags of water availability in a simpler way than if we had over specified the model with an explicit month term. Finally, the DTW model also included a test of beaver influence as a factorial term. We felt PDO is an at least decadal phenomenon, and we determined its finest scale time step of monthly was too coarse to apply in the DTW model. The total number of observations for DTW models was 942 with the nonlinear period of record trend based on 21 years.

We model the effect of months within each year's growing season as a nonlinear or smoothed term in each climate, water balance and hydrologic GAM (note that SWE has only one value per year and so we cannot do this with this response). We restrict the complexity of this smooth to avoid overfitting. While a smooth on months does not explicitly model a month effect of test for independence between months, it provides a flexible non-linear fit to seasonal (monthly) trends. This helps model shared seasonal structure by accounting for patterns across months, reducing unexplained variability, and, in effect, isolating the estimate of an overall trend across the period of record (the primary goal of these models) from the within year variability. Likewise, the random effect used for years across the period of record term helps model variability across years non-linearly by treating years as a random grouping factor. This approach has several benefits. It captures unmeasured factors influencing the response that vary across years (e.g., climate anomalies or environmental conditions) and by modeling year-to-year variability, the term accounts for correlations induced by shared year-level factors, which can help approximate independence across years. We present partial residual plots in this Supplemental's Results section to help visualize individual model terms and their fits.

Models for total and mean pond size in each year could only use a single predictor for years (as a factor). Effective sample size for total and mean pond size was seven as summarized from 2401 patches into total areas and mean pond size.

We report all p-values and interpret as statistically significant any result as meaningful if its p value is below or equal to 0.05. However, test results that are between 0.05 and ~0.10 are still likely meaningful and we interpret these marginal cases accordingly.

We visually examine autocorrelation and partial autocorrelations via the R autocorrelation functions ACF() and PACF() and then test for an effect using a Ljung and Box test (Ljung and Box 1978) with each lag set based on the model's sample size. If this test indicates that residuals were autocorrelated we further examined the ACF and partial ACF plots. We do not expect model results are strongly influenced even with a significant Ljung and Box test if there was not a strong suggestion of autocorrelation in the residuals as shown in these plots. We include partial plots in this Supplemental's Results section when there is clear evidence of a likely meaningful autocorrelation.

Confidence intervals (95%) were estimated around each linear trend's coefficient. For non-linear trends, confidence intervals were pointwise estimates to allow the intervals to follow the variability of a smooth.

Nonparametric models of total pond and willow patch area

Differences in median tall and short willow patch size between 1999 and 2019 were assessed with non-parametric Kruskal Wallis tests from the base stats package in R as these variables were strongly non-normal. Sample size for willow patch models ranged from 199 to 454 depending on height class and inside/outside the park. To test for differences in total area across only two periods, we used a permutation test to generate a null distribution of differences in sums expected under the assumption of no true difference between the periods. We then calculated an exact probability as the sum of all differences in sums greater than the observed difference divided by the number of permutations.

Vegetation metrics

Total absolute non-native invasive taxa cover

Nativity describes whether a species is found within its area of evolutionary origin and/or arrived without human intervention. Nonnative species are often introduced through intentional or unintentional human action (Pysek et al. 2004, Fertig 2011). Nonnative species can be invasive and can have undesirable effects on ecosystem function (Byers et al. 2002, Levine et al. 2003, Fridley et al. 2007). They have been linked to reduced overall species diversity (Meiners et al. 2001), altered resource dynamics (Ehrenfeld 2003), and shifted interactions between species (Christian and Wilson 1999). Nonnative species are often the focus of park management of vegetation, including at RMNP. We created an index of the degree of invasion by weighting the average of the relative cover of taxa by coefficients of invasiveness ("I-ranks") as developed by Morse et al. (2004). I-ranks are scored based on the ability of a species to change ecosystem processes; invade relatively undisturbed ecological communities; disperse to new areas readily; and cause substantial impacts on rare or vulnerable species or ecological communities, or high-quality examples of more common communities.

Conservatism

Conservatism describes a species' fidelity to a specific habitat or range of environmental conditions absent of human disturbance (Wilhelm and Ladd 1988, Herman et al. 1997, Matthews et al. 2015). Anthropogenic impacts can cause dramatic shifts in ecological processes and habitat conditions and push disturbance regimes outside a natural range of intensity, frequency, and duration. More conservative species are not able to quickly respond to such rapid alterations compared to broad-niche generalists and are often the first to disappear from habitats heavily impacted by human activities. The composition of conservative species at a particular site integrates spatial and temporal

impacts and can serve as an indicator of ecological integrity or condition. To assess conservatism, we use metrics based on "coefficients of conservatism" (also known as "C-scores") assigned to the flora of Colorado by a panel of experts following the methods described by Swink and Wilhelm (1994). Colorado C-scores were originally assigned in 2007 (Rocchio 2007b) and updated in 2020 (Smith et al. 2020). C-scores range from 0 to 10 and represent the estimated probability that a plant shows high fidelity to landscapes relatively unaltered from pre-European settlement conditions. Nonnative species are given C-scores of 0 by default. Low C-values are assigned to species that demonstrate little fidelity to unaltered landscapes or have wide ecological tolerances and may be found almost anywhere. High C-values are assigned to species only found in high quality natural areas and that cannot tolerate habitat degradation. Using the C-scores of all or select species present within a site, a suite of metrics can be calculated that convey different aspects of the site's condition and disturbance history. We estimated the conservatism of each sample by averaging cover weighted conservatism scores for all taxa.

Wetland affinity

Wetland affinity measures the prevalence of species in a community that have a demonstrated ability because of morphological or physiological adaptations and/or reproductive strategies to achieve maturity and reproduce in wetlands. Higher wetland affinity in a sample indicates that vegetation composition includes a higher proportion of wetland obligates and thus likely higher and/or more stable water tables. Because the mix of species at a site responds over time and space to variation in hydrologic regime, wetland affinity integrates seasonal and annual fluctuations in groundwater levels (Grace et al. 2012). Wetland affinity is likely a useful, integrated proxy for more complex and expensive measures of groundwater hydrologic regime (Loheide and Gorelick 2007). For wetland affinity, we estimated a mean cover weighted score across all taxa in a sample.

Composition and structure

Multivariate vegetation composition analyses were conducted using vegan 2.6-4 (Oskanen et al. 2022) and the R vegan library (Oksanen et al. 2024). Wetland community types were first identified with hierarchical agglomerative cluster analysis using Sorenson distance and flexible beta linkages less than or equal to -0.25. Indicator species analysis (Dufrêne and Legendre 1997) identified species characterizing each cluster and a Monte Carlo analysis was applied to understand the significance of species' indicator values within each cluster. Indicator species analysis was used to prune the dendrogram and optimize the number of clusters (McCune and Mefford 2018). We averaged p-values across all species for each cluster level using Monte Carlo analysis and chose the cluster with the lowest average P value. Final site type assignment in each year and indicator species were reviewed and adjusted based on our long-term experience in the system. Non-metric multidimensional scaling (NMDS) was used to determine community dissimilarity across years and wetland community types. Analysis of similarities (ANOSIM) were used to test the significance of the difference in species composition among community types across and within years. NMDS paired with vector fitting (function 'envfit' in vegan) was used to examine the relationships between overall community composition and total absolute non-native invasive taxa cover, mean conservatism score, and mean wetland affinity score. To visualize and describe community composition changes across years and wetland types, a single NMDS was used. To determine which summary vegetation metrics (total absolute non-native invasive taxa cover, mean conservatism score, and mean wetland affinity score were predictive of community composition in each sample year, two NMDS with two corresponding PERMANOVAs were run, each with 1000 permutations based on Euclidean distances. Total number of observations for the ordination was 784 (number of vegetation records across both 1998 and 2021).

Appendix S3: Climate and water balance results

Climate change is creating stress and potential ecosystem collapses around the world (Ling and Keane 2024), including riparian ecosystems in western US national parks (Gonzalez et al. 2018, Thoma et al. 2020, Carroll et al. 2024). Rocky Mountain glaciers are disappearing (Hall and Fagre 2003) altering streams (McKernan et al. 2018), large wildfires are more common (Dennison, et al. 2014), and declining snowpacks (Mote et al. 2018) are melting earlier in spring (Milly and Dunne 2020, Musselmann et al. 2021). These changes may be increasing the length of the snow-free season, and the potential for increased temperature and evapotranspiration that can make modest droughts into extreme ones (Overpeck 2013). At a regional scale the Colorado River basin has been under extreme drought conditions for more than 20 years (Cook et al. 2015, Williams et al. 2022, Gangopadhyay et al. 2022), with reservoirs reaching record lows, and water delivery to urban and agricultural areas being compromised.

We found consistent evidence of warming temperatures, decreased summer precipitation, increasing climatic water deficit, marginally reduced runoff, and increasing potential evapotranspiration in the KV. However, while ground water elevations varied with the influence of beaver dams, in general DTW changed little from 1996 to 2023. Beaver dams can create surface and subsurface hydrological connectivity and maintain high and stable water tables (Westbrook et al. 2006, Karran et al. 2018) as historically occurred in the KV. Median growing season Colorado River flow was statistically unchanged from 1953 to 2023. This contrasts with patterns of discharge in many other locations in the Colorado River Basin where flows have declined (Udall and Overpeck 2017). While lower elevation regions in the Colorado River basin have experienced hydrologic changes, the KV watershed with its extensive cold alpine and subalpine regions may have at least short-term buffering from increasing temperatures and function as a hydrological refugia for stream and ground water supported ecosystems. The snowmelt dominated hydrologic regime is a key stabilizing factor, as it is in the Canadian Rocky Mountains (Harder et al. 2015), and severe drought has been uncommon in the past few decades.

Mountain pine beetle and spruce beetle caused large-scale lodgepole pine and Engelman spruce mortality in the KV watershed in recent decades (Perovich and Sibold 2016, Potter and Conkling 2016, Carlson et al. 2021). Watersheds with beetle killed forests had 24% less snow sublimation, an increase in snow duration into the summer (Frank et al. 2019) and a 28-36% decrease in annual evapotranspiration (Frank et al. 2014) all of which may be increasing water availability in the KV. Forest dieback should increase water availability but in the absence of beaver the KV water table has remained stable. As forests regenerate or temperature increases, water availability could be reduced.

Table S.1 provides summary statistics for climate and water balance models. Figures S.1 to S.6 provide R output from GAM models of climate and WB parameters.

Table S.1. Confidence intervals and autocorrelation results for period of record for climate and water balance trend models.

Model	POR trend coefficient lower 95% CI	POR trend coefficient upper 95% CI	Lung Box autocorrelation test (chi-square statistic, approx.. degrees of freedom and p-value)	Autocorrelation (A/PACF) visual interpretation
Median growing season monthly air temperature, C	0.63	0.94	X-squared = 42.97, df = 20, p-value = 0.002	Little evidence of lags
Total growing season monthly precipitation as rain, mm	-7.64	-0.82	X-squared = 22, df = 20, p-value = 0.3	No evidence of lags
Maximum annual week of April 1 snow water equivalency (mm)	-83.20	2.00	X-squared = 29, df = 20, p-value = 0.08	No evidence of lags
Total growing season monthly climatic deficit (mm)	0.07	2.19	X-squared = 54, df = 20, p-value = 0.00005	Some evidence of lags
Total growing season monthly cumulative runoff (mm),	-11.4	3.15	X-squared = 79, df = 20, p-value = 0.000000006	Little evidence of lags
Total growing season monthly potential evapotranspiration (mm)	0.70	2.20	X-squared = 17, df = 20, p-value = 0.6	No evidence of lags

```
>  anova(gn, g1, g2p, g2)
Analysis of Deviance Table

Model 1: response ~ 1
Model 2: response ~ scaled_date_por
Model 3: response ~ scaled_date_por + s(scaled_date_doy, k = 5) + s(yearF,
    bs = "re") + PDO
Model 4: response ~ scaled_date_por + s(scaled_date_doy, k = 5) + s(yearF,
    bs = "re")
  Resid. Df Resid. Dev      Df Deviance        F Pr(>F)
1    366.00     3175.5
2    365.00     2951.2  1.0000   224.24 227.2394 <2e-16 ***
3    301.72      315.3 63.2758  2635.92  42.2145 <2e-16 ***
4    302.37      315.4 -0.6409    -0.13   0.2093 0.5376
---
Signif. codes:  0 ‘***’ 0.001 ‘**’ 0.01 ‘*’ 0.05 ‘.’ 0.1 ‘ ’ 1
>   AIC(gn, g1, g2p, g2)
          df      AIC
gn   2.00000 1837.431
g1   3.00000 1812.554
g2p 48.48739 1082.749
g2  47.76562 1081.460
>   summary(g2)

Family: gaussian
Link function: identity

Formula:
response ~ scaled_date_por + s(scaled_date_doy, k = 5) + s(yearF,
    bs = "re")

Parametric coefficients:
                Estimate Std. Error t value Pr(>|t|)
(Intercept)     11.09192    0.07911  140.21   <2e-16 ***
scaled_date_por  0.78980    0.07945    9.94   <2e-16 ***
---
Signif. codes:  0 ‘***’ 0.001 ‘**’ 0.01 ‘*’ 0.05 ‘.’ 0.1 ‘ ’ 1

Approximate significance of smooth terms:
                      edf Ref.df       F  p-value
s(scaled_date_doy)  3.705  3.948 637.397  < 2e-16 ***
s(yearF)           41.060 73.000   1.309 4.65e-07 ***
---
Signif. codes:  0 ‘***’ 0.001 ‘**’ 0.01 ‘*’ 0.05 ‘.’ 0.1 ‘ ’ 1

R-sq.(adj) =  0.886   Deviance explained = 90.1%
GCV = 1.1288  Scale est. = 0.985     n = 367
```

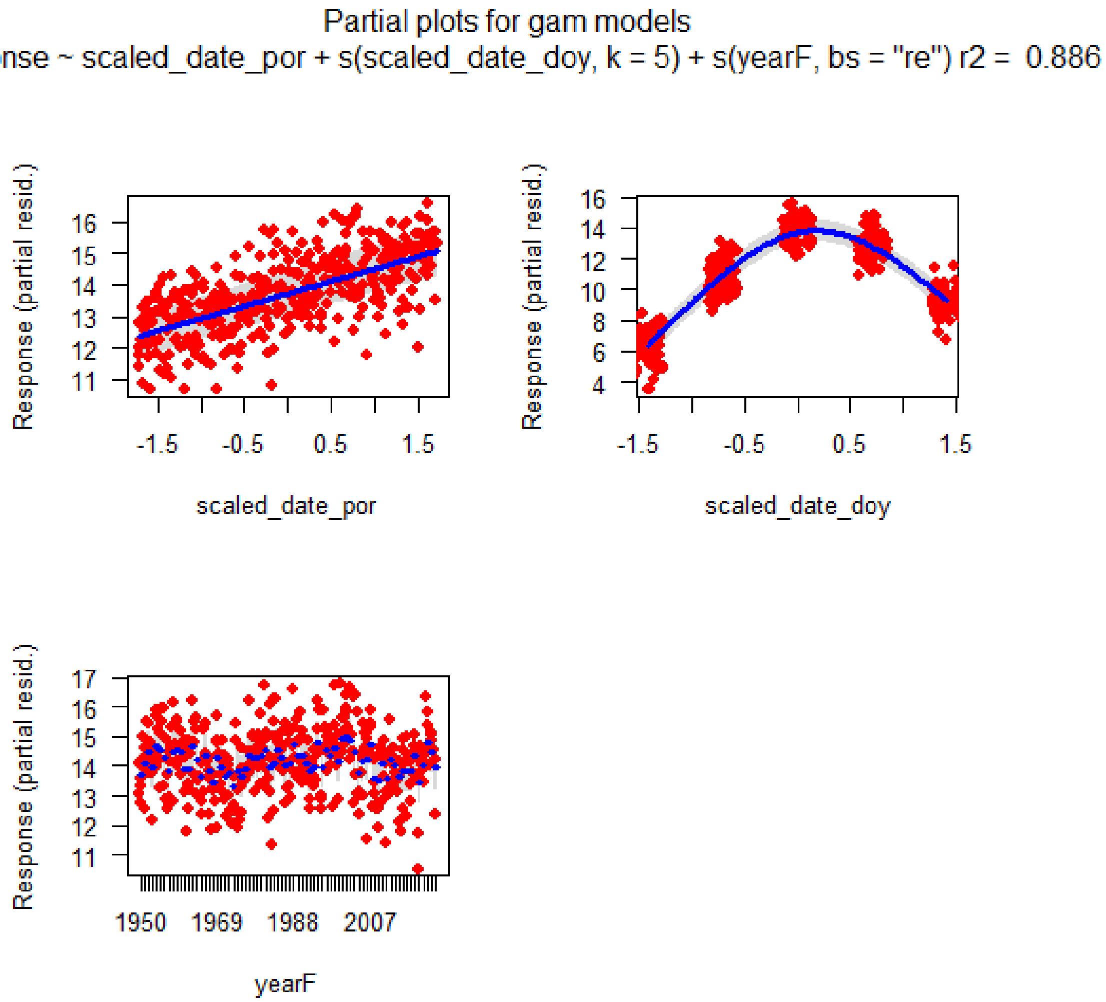

Figure S.1. (A) Model selection and General Additive Model results for median growing season monthly air temperature; (B) partial residual plots for final model of median growing season monthly air temperature.

```
>  anova(gn, g1, g2p, g2)
Analysis of Deviance Table

Model 1: response ~ 1
Model 2: response ~ scaled_date_por
Model 3: response ~ scaled_date_por + s(scaled_date_doy, k = 5) + s(yearF,
    bs = "re") + PDO
Model 4: response ~ scaled_date_por + s(scaled_date_doy, k = 5) + s(yearF,
    bs = "re")
  Resid. Df Resid. Dev      Df Deviance       F    Pr(>F)
1    369.00     279643
2    368.00     273063  1.0000     6579 10.7485  0.001161 **
3    316.92     205514 51.0768    67550  2.1606 3.297e-05 ***
4    313.88     203270  3.0441     2243  1.2039  0.308551
---
Signif. codes:  0 ‘***’ 0.001 ‘**’ 0.01 ‘*’ 0.05 ‘.’ 0.1 ‘ ’ 1
>   AIC(gn, g1, g2p, g2)
          df      AIC
gn   2.00000 3506.288
g1   3.00000 3499.478
g2p 36.46761 3461.263
g2  38.92072 3462.108
>   summary(g2)

Family: gaussian
Link function: identity

Formula:
response ~ scaled_date_por + s(scaled_date_doy, k = 5) + s(yearF,
    bs = "re")

Parametric coefficients:
                Estimate Std. Error t value Pr(>|t|)
(Intercept)       45.399      1.736  26.157   <2e-16 ***
scaled_date_por   -4.231      1.738  -2.434   0.0155 *
---
Signif. codes:  0 ‘***’ 0.001 ‘**’ 0.01 ‘*’ 0.05 ‘.’ 0.1 ‘ ’ 1

Approximate significance of smooth terms:
                      edf Ref.df     F  p-value
s(scaled_date_doy)  3.462  3.836 4.789 0.000752 ***
s(yearF)           32.459 73.000 0.810 0.000234 ***
---
Signif. codes:  0 ‘***’ 0.001 ‘**’ 0.01 ‘*’ 0.05 ‘.’ 0.1 ‘ ’ 1

R-sq.(adj) =  0.192   Deviance explained = 27.3%
GCV = 682.01  Scale est. = 612.11    n = 370
```

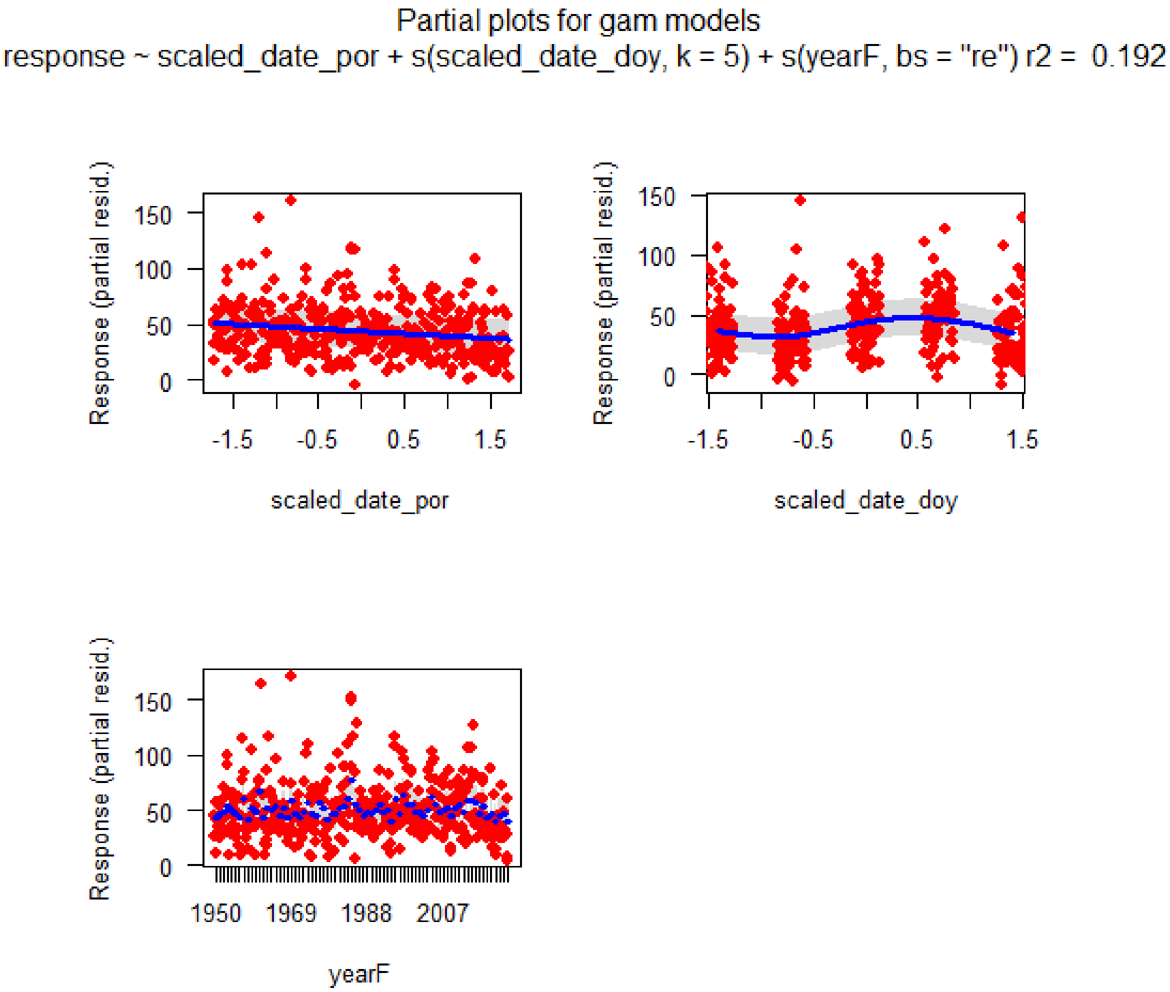

Figure S.2. (A) Model selection and General Additive Model results for total growing season monthly precipitation; (B) partial residual plots for final model of total growing season monthly precipitation.

```
>   anova(gn, g1, g2p, g2)
Analysis of Deviance Table

Model 1: response ~ 1
Model 2: response ~ scaled_date_por
Model 3: response ~ scaled_date_por + PDO
Model 4: response ~ scaled_date_por
  Resid. Df Resid. Dev Df Deviance      F  Pr(>F)
1        43     942070
2        42     869792  1    72278 3.5551 0.06646 .
3        41     833559  1    36234 1.7822 0.18924
4        42     869792 -1   -36234 1.7822 0.18924
---
Signif. codes:  0 ‘***’ 0.001 ‘**’ 0.01 ‘*’ 0.05 ‘.’ 0.1 ‘ ’ 1
>   AIC(gn, g1, g2p, g2)
    df      AIC
gn   2 567.6190
g1   3 566.1067
g2p  4 566.2345
g2   3 566.1067
>   summary(g2)

Family: gaussian
Link function: identity

Formula:
response ~ scaled_date_por

Parametric coefficients:
                Estimate Std. Error t value Pr(>|t|)
(Intercept)       626.09      21.70  28.857   <2e-16 ***
scaled_date_por   -40.62      21.75  -1.868   0.0687 .
---
Signif. codes:  0 ‘***’ 0.001 ‘**’ 0.01 ‘*’ 0.05 ‘.’ 0.1 ‘ ’ 1


R-sq.(adj) =  0.0547   Deviance explained = 7.67%
GCV =  21695  Scale est. = 20709     n = 44
```

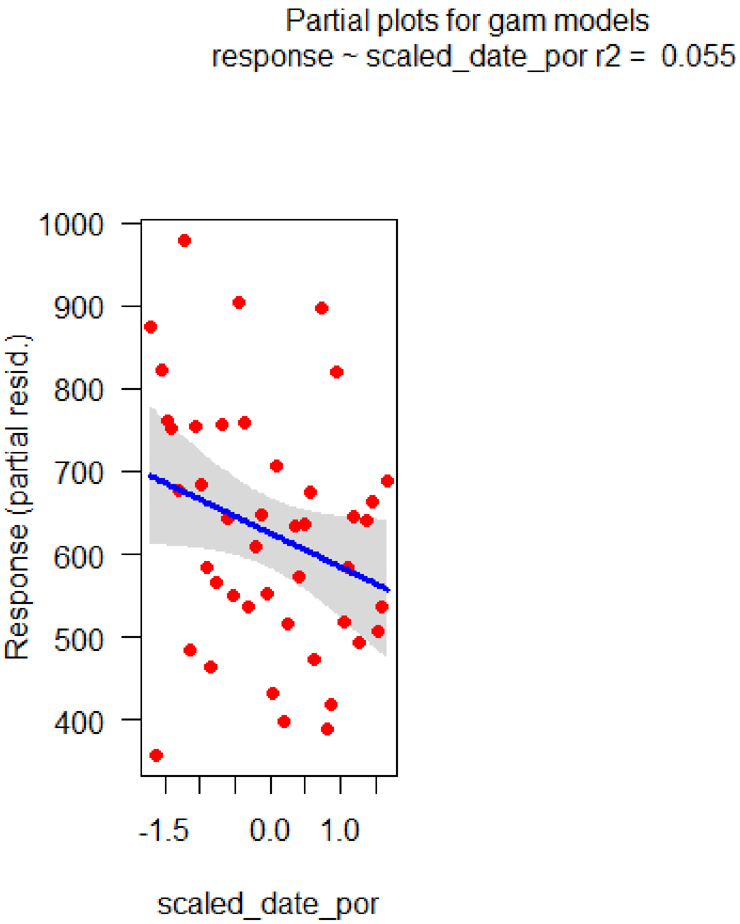

Figure S.3. (A) Model selection and General Additive Model results for Maximum annual week of April 1 snow water equivalency; (B) partial residual plots for final model of April 1 snow water equivalency.

```
>   anova(gn, g1, g2p, g2)
Analysis of Deviance Table

Model 1: response ~ 1
Model 2: response ~ scaled_date_por
Model 3: response ~ scaled_date_por + s(scaled_date_doy, k = 5) + s(yearF,
    bs = "re") + PDO
Model 4: response ~ scaled_date_por + s(scaled_date_doy, k = 5) + s(yearF,
    bs = "re")
  Resid. Df Resid. Dev       Df Deviance       F    Pr(>F)
1    219.00     9484.4
2    218.00     9191.1  1.00000    293.3 20.5818 1.063e-05 ***
3    173.50     2582.2 44.50134   6608.9 10.4210 < 2.2e-16 ***
4    174.22     2606.8 -0.71735    -24.6  2.4031     0.129
---
Signif. codes:  0 ‘***’ 0.001 ‘**’ 0.01 ‘*’ 0.05 ‘.’ 0.1 ‘ ’ 1
>   AIC(gn, g1, g2p, g2)
          df      AIC
gn   2.00000 1456.364
g1   3.00000 1451.453
g2p 39.80577 1245.754
g2  39.16328 1246.552
>   summary(g2)

Family: gaussian
Link function: identity

Formula:
response ~ scaled_date_por + s(scaled_date_doy, k = 5) + s(yearF,
    bs = "re")

Parametric coefficients:
                Estimate Std. Error t value Pr(>|t|)
(Intercept)       8.1249     0.5406   15.03   <2e-16 ***
scaled_date_por   1.1324     0.5419    2.09    0.038 *
---
Signif. codes:  0 ‘***’ 0.001 ‘**’ 0.01 ‘*’ 0.05 ‘.’ 0.1 ‘ ’ 1

Approximate significance of smooth terms:
                      edf Ref.df      F p-value
s(scaled_date_doy)  3.528  3.872 70.930  <2e-16 ***
s(yearF)           32.636 43.000  3.404  <2e-16 ***
---
Signif. codes:  0 ‘***’ 0.001 ‘**’ 0.01 ‘*’ 0.05 ‘.’ 0.1 ‘ ’ 1

R-sq.(adj) =  0.669   Deviance explained = 72.5%
GCV = 17.344  Scale est. = 14.336    n = 220
```

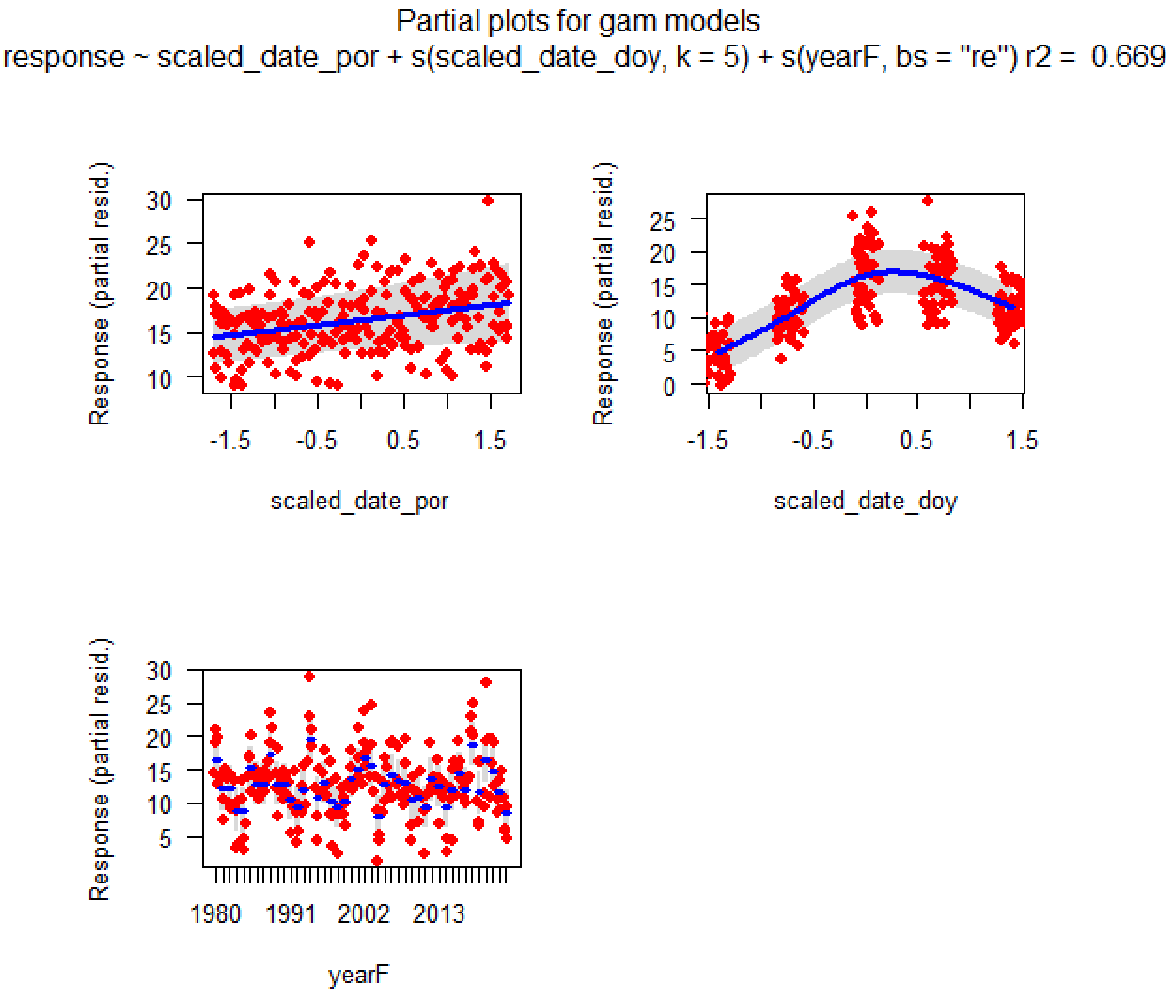

Figure S.4. (A) Model selection and General Additive Model results for total growing season monthly climatic deficit; (B) partial residual plots for final model of total growing season monthly climatic deficit.

```
>   anova(gn, g1, g2p, g2)
Analysis of Deviance Table

Model 1: response ~ 1
Model 2: response ~ scaled_date_por
Model 3: response ~ scaled_date_por + s(scaled_date_doy, k = 5) + s(yearF,
    bs = "re") + PDO
Model 4: response ~ scaled_date_por + s(scaled_date_doy, k = 5) + s(yearF,
    bs = "re")
  Resid. Df Resid. Dev     Df Deviance     F Pr(>F)
1       219     812286
2       218     807774  1.000     4512  5.02  0.026 *
3       175     165552 42.830   642222 16.69 <2e-16 ***
4       176     168586 -0.892    -3034  3.78  0.058 .
---
Signif. codes:  0 ‘***’ 0.001 ‘**’ 0.01 ‘*’ 0.05 ‘.’ 0.1 ‘ ’ 1
>   AIC(gn, g1, g2p, g2)
      df  AIC
gn   2.0 2435
g1   3.0 2436
g2p 36.7 2155
g2  35.7 2157
>   summary(g2)

Family: gaussian
Link function: identity

Formula:
response ~ scaled_date_por + s(scaled_date_doy, k = 5) + s(yearF,
    bs = "re")

Parametric coefficients:
                Estimate Std. Error t value Pr(>|t|)
(Intercept)        40.83       3.71   11.01   <2e-16 ***
scaled_date_por    -4.14       3.72   -1.11     0.27
---
Signif. codes:  0 ‘***’ 0.001 ‘**’ 0.01 ‘*’ 0.05 ‘.’ 0.1 ‘ ’ 1

Approximate significance of smooth terms:
                     edf Ref.df      F p-value
s(scaled_date_doy)  3.29   3.73 152.67  <2e-16 ***
s(yearF)           29.38  43.00   2.27  <2e-16 ***
---
Signif. codes:  0 ‘***’ 0.001 ‘**’ 0.01 ‘*’ 0.05 ‘.’ 0.1 ‘ ’ 1

R-sq.(adj) =  0.755   Deviance explained = 79.2%
GCV = 1079.9  Scale est. = 909.68    n = 220
```

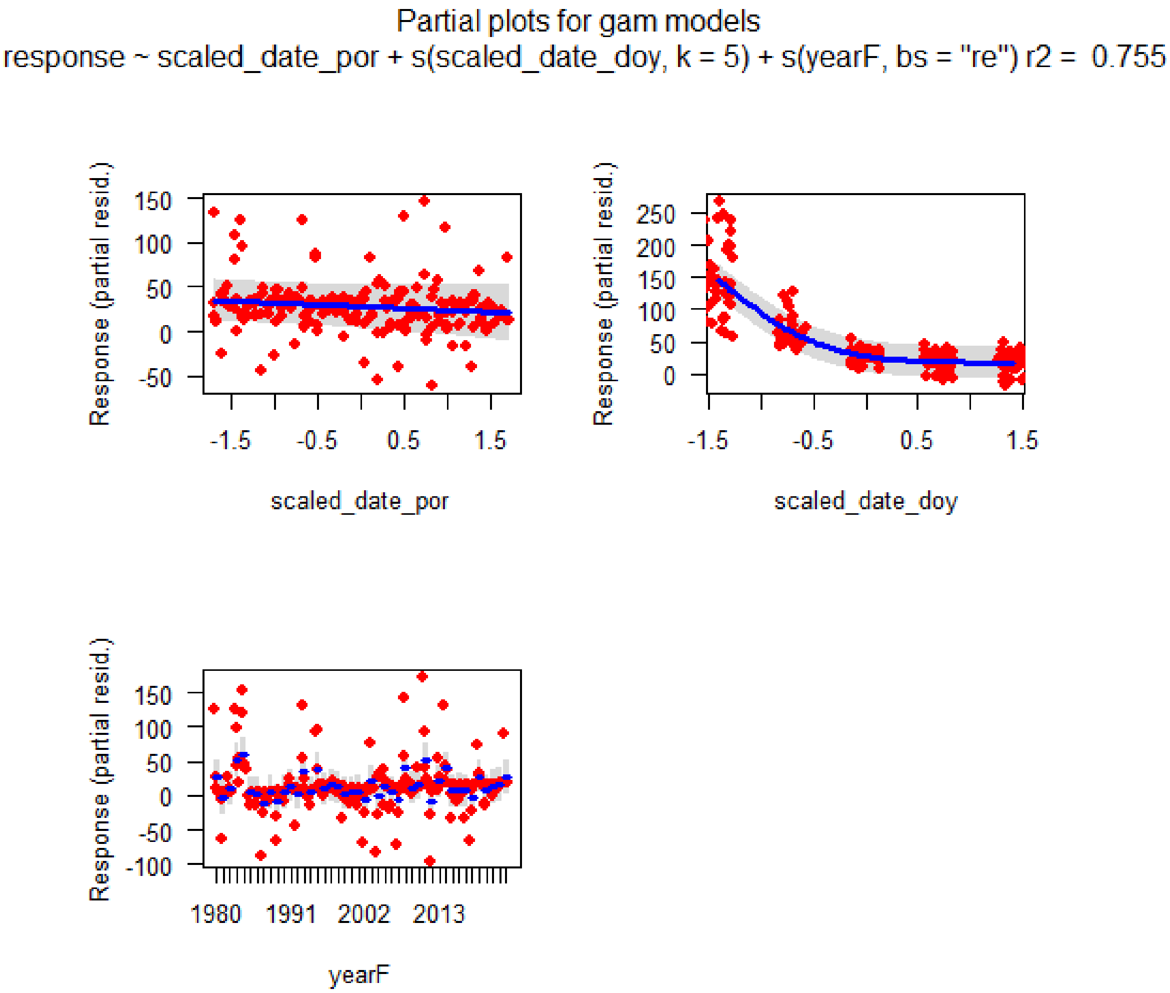

Figure S.5. (A) Model selection and General Additive Model results for total growing season monthly cumulative runoff; (B) partial residual plots for final model of total growing season monthly cumulative runoff.

```
>   anova(gn, g1, g2p, g2)
Analysis of Deviance Table

Model 1: response ~ 1
Model 2: response ~ scaled_date_por
Model 3: response ~ scaled_date_por + s(scaled_date_doy, k = 5) + s(yearF,
    bs = "re") + PDO
Model 4: response ~ scaled_date_por + s(scaled_date_doy, k = 5) + s(yearF,
    bs = "re")
  Resid. Df Resid. Dev    Df Deviance     F   Pr(>F)
1       219      47467
2       218      47000  1.00      467 20.53 0.00001 ***
3       195       4628 23.14    42372 80.50 < 2e-16 ***
4       193       4597  1.67       31  0.82    0.42
---
Signif. codes:  0 ‘***’ 0.001 ‘**’ 0.01 ‘*’ 0.05 ‘.’ 0.1 ‘ ’ 1
>   AIC(gn, g1, g2p, g2)
      df  AIC
gn   2.0 1811
g1   3.0 1810
g2p 18.1 1331
g2  18.9 1331
>   summary(g2)

Family: gaussian
Link function: identity

Formula:
response ~ scaled_date_por + s(scaled_date_doy, k = 5) + s(yearF,
    bs = "re")

Parametric coefficients:
                Estimate Std. Error t value Pr(>|t|)
(Intercept)       50.480      0.382  132.21   <2e-16 ***
scaled_date_por    1.449      0.383    3.79   0.0002 ***
---
Signif. codes:  0 ‘***’ 0.001 ‘**’ 0.01 ‘*’ 0.05 ‘.’ 0.1 ‘ ’ 1

Approximate significance of smooth terms:
                     edf Ref.df     F p-value
s(scaled_date_doy)  3.65   3.93 462.2  <2e-16 ***
s(yearF)           12.22  43.00   0.4   0.062 .
---
Signif. codes:  0 ‘***’ 0.001 ‘**’ 0.01 ‘*’ 0.05 ‘.’ 0.1 ‘ ’ 1

R-sq.(adj) =  0.895   Deviance explained = 90.3%
GCV = 24.752  Scale est. = 22.742    n = 220
```

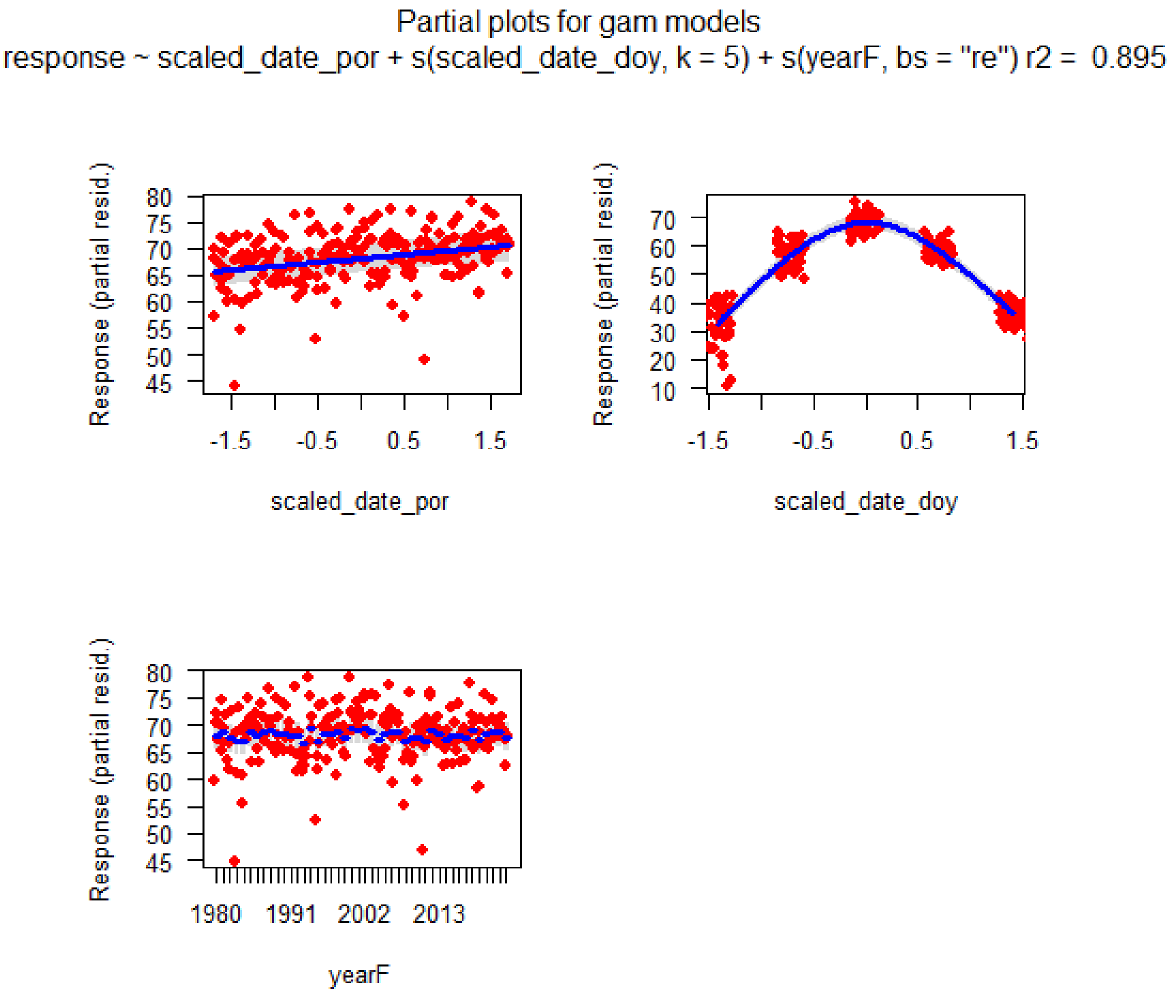

Figure S.6. (A) Model selection and General Additive Model results for total growing season monthly potential evapotranspiration; (B) partial residual plots for final model of total growing season monthly potential evapotranspiration.

Appendix S4: Surface water results

Colorado River and Grand River Ditch flow

Table S.2 provides summary statistics for stream flow models. Figure S.9 to S.11 provide R output from GAM models of stream flow variables.

Table S.2. Confidence intervals and autocorrelation results for period of record surface water flow trend models.

Model	POR trend coefficient lower 95% CI	POR trend coefficient upper 95% CI	Lung Box autocorrelation test (chi-square statistic, approx.. degrees of freedom and p-value)	Autocorrelation (A/PACF) visual interpretation
Mean growing season monthly Colorado River flow, m3/second	-0.18	0.63	X-squared = 44, df = 20, p-value = 0.001	Little evidence of lags
Variability in growing season monthly Colorado River flow	-0.03	0.01	X-squared = 21, df = 20, p-value = 0.4	No evidence of lags
Proportion of growing season monthly flow in the Colorado River versus the Grand River Ditch	-0.013	0.023	X-squared = 47, df = 20, p-value = 0.0005	Little evidence of lags

```
>   anova(gn, g1, g2p, g2)
Analysis of Deviance Table

Model 1: response ~ 1
Model 2: response ~ scaled_date_por
Model 3: response ~ scaled_date_por + s(scaled_date_doy, k = 5) + s(yearF, 
    bs = "re") + PDO
Model 4: response ~ scaled_date_por + s(scaled_date_doy, k = 5) + s(yearF, 
    bs = "re")
  Resid. Df Resid. Dev        Df Deviance       F Pr(>F)    
1    353.00     5961.4                                        
2    352.00     5946.7  1.000000     14.8  2.5888 0.1087    
3    290.94     1756.0 61.062774   4190.6 12.0281 <2e-16 ***
4    290.96     1755.2 -0.023849      0.8                     
---
Signif. codes:  0 ‘***’ 0.001 ‘**’ 0.01 ‘*’ 0.05 ‘.’ 0.1 ‘ ’ 1
>   AIC(gn, g1, g2p, g2)
          df      AIC
gn   2.00000 2008.222
g1   3.00000 2009.344
g2p 47.22687 1666.001
g2  47.24652 1665.872
>   summary(g2)

Family: gaussian 
Link function: identity 

Formula:
response ~ scaled_date_por + s(scaled_date_doy, k = 5) + s(yearF, 
    bs = "re")

Parametric coefficients:
                Estimate Std. Error t value Pr(>|t|)    
(Intercept)       3.7906     0.1973   19.21   <2e-16 ***
scaled_date_por   0.2241     0.2056    1.09    0.277    
---
Signif. codes:  0 ‘***’ 0.001 ‘**’ 0.01 ‘*’ 0.05 ‘.’ 0.1 ‘ ’ 1

Approximate significance of smooth terms:
                      edf Ref.df       F p-value    
s(scaled_date_doy)  3.971  3.999 148.062  <2e-16 ***
s(yearF)           40.276 70.000   1.386  <2e-16 ***
---
Signif. codes:  0 ‘***’ 0.001 ‘**’ 0.01 ‘*’ 0.05 ‘.’ 0.1 ‘ ’ 1

R-sq.(adj) =  0.662   Deviance explained = 70.6%
GCV = 6.5603  Scale est. = 5.7033    n = 354
```

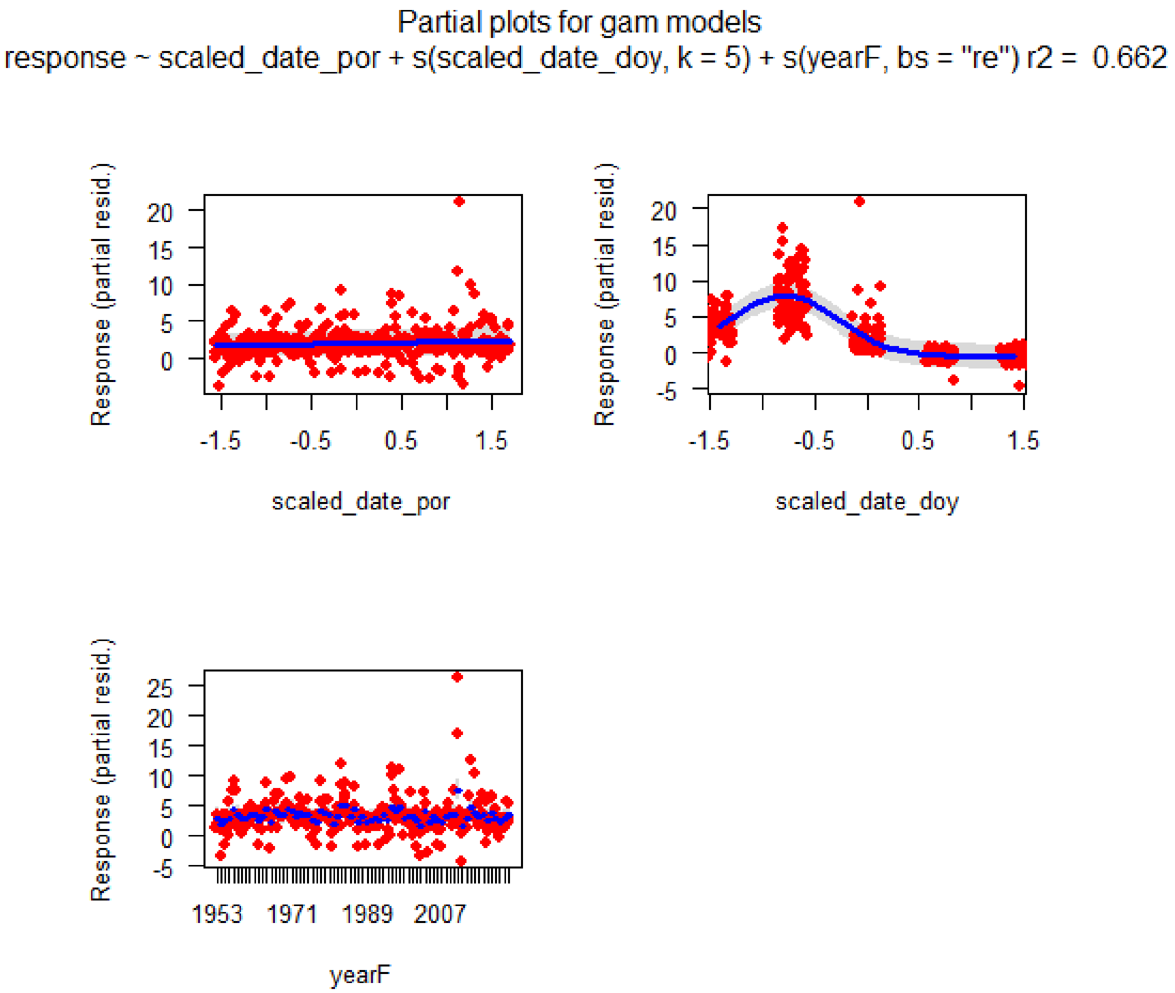

Figure S.7. (A) Model selection and General Additive Model results for median growing season monthly Colorado River flow; (B) partial residual plots for final model of median growing season monthly Colorado River flow.

```
>   anova(gn, g1, g2p, g2)
Analysis of Deviance Table

Model 1: response ~ 1
Model 2: response ~ scaled_date_por
Model 3: response ~ scaled_date_por + s(scaled_date_doy, k = 5) + s(yearF,
    bs = "re") + PDO
Model 4: response ~ scaled_date_por + s(scaled_date_doy, k = 5) + s(yearF,
    bs = "re")
  Resid. Df Resid. Dev      Df Deviance       F Pr(>F)
1       353    14.8295
2       352    14.8162  1.0000   0.0133  0.5070 0.4769
3       347     9.1142  4.9982   5.7020 43.4402 <2e-16 ***
4       348     9.1635 -1.0000  -0.0493  1.8763 0.1716
---
Signif. codes:  0 ‘***’ 0.001 ‘**’ 0.01 ‘*’ 0.05 ‘.’ 0.1 ‘ ’ 1
>   AIC(gn, g1, g2p, g2)
          df       AIC
gn  2.000000 -114.5195
g1  3.000000 -112.8375
g2p 7.946588 -274.9488
g2  6.946034 -275.0412
>   summary(g2)

Family: gaussian
Link function: identity

Formula:
response ~ scaled_date_por + s(scaled_date_doy, k = 5) + s(yearF,
    bs = "re")

Parametric coefficients:
                 Estimate Std. Error t value Pr(>|t|)
(Intercept)      0.388617   0.008650  44.925   <2e-16 ***
scaled_date_por -0.006797   0.009026  -0.753    0.452
---
Signif. codes:  0 ‘***’ 0.001 ‘**’ 0.01 ‘*’ 0.05 ‘.’ 0.1 ‘ ’ 1

Approximate significance of smooth terms:
                         edf Ref.df     F p-value
s(scaled_date_doy) 3.946e+00  3.998 53.06  <2e-16 ***
s(yearF)           2.474e-07 70.000  0.00   0.582
---
Signif. codes:  0 ‘***’ 0.001 ‘**’ 0.01 ‘*’ 0.05 ‘.’ 0.1 ‘ ’ 1

R-sq.(adj) =  0.373   Deviance explained = 38.2%
GCV = 0.026777  Scale est. = 0.026328  n = 354
```

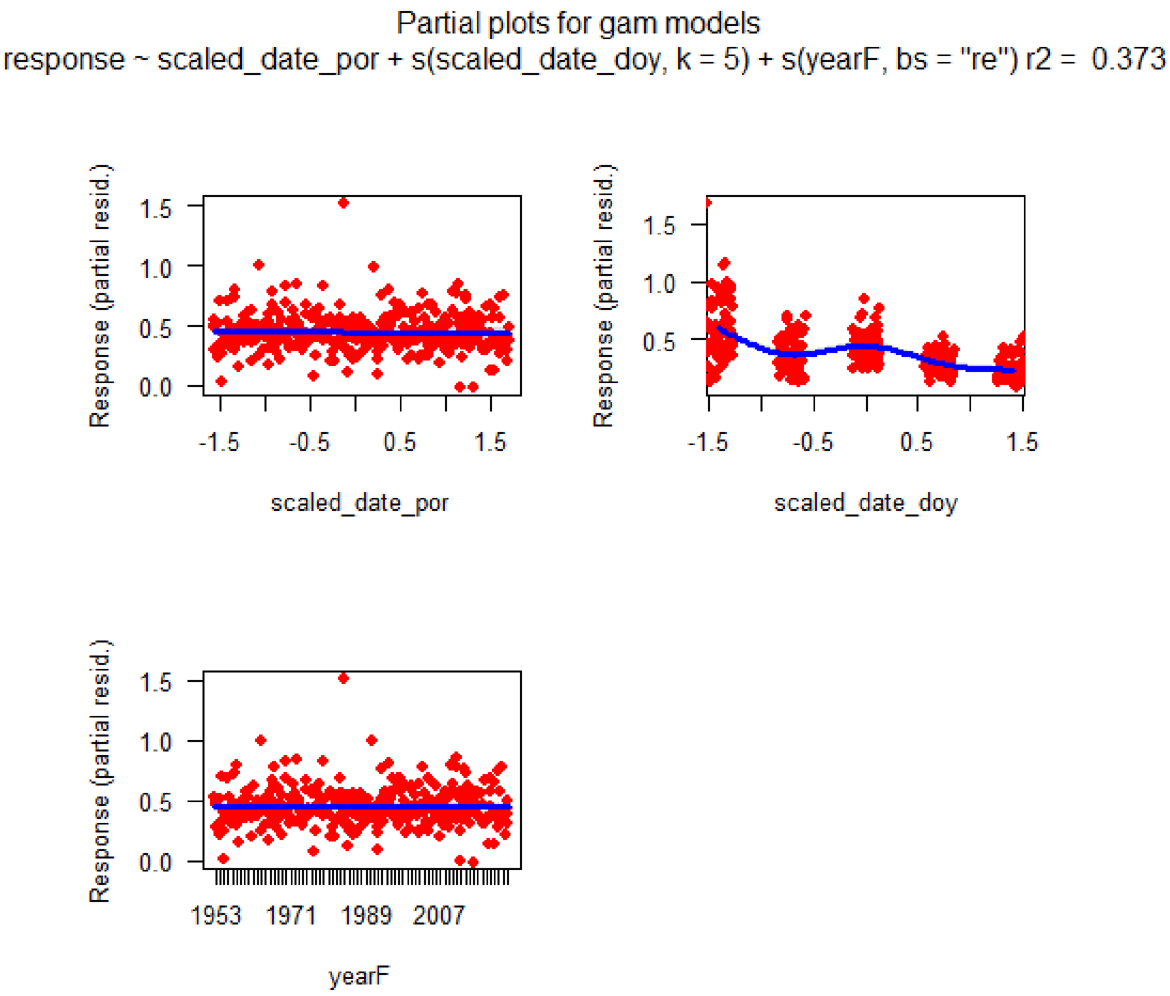

Figure S.8. (A) Model selection and General Additive Model results for variability in growing season monthly Colorado River flow; (B) partial residual plots for final model of variability in growing season monthly Colorado River flow.

```
>   anova(gn, g1, g2p, g2)
Analysis of Deviance Table

Model 1: response ~ 1
Model 2: response ~ scaled_date_por
Model 3: response ~ scaled_date_por + s(scaled_date_doy, k = 5) + s(yearF, 
    bs = "re") + PDO
Model 4: response ~ scaled_date_por + s(scaled_date_doy, k = 5) + s(yearF, 
    bs = "re")
  Resid. Df Resid. Dev       Df Deviance       F Pr(>F)    
1    353.00    15.5671                                     
2    352.00    15.5616  1.00000   0.0055  0.3377 0.5616    
3    304.11     5.2229 47.88651  10.3387 13.2593 <2e-16 ***
4    305.01     5.2332 -0.89323  -0.0102  0.7016 0.3877    
---
Signif. codes:  0 ‘***’ 0.001 ‘**’ 0.01 ‘*’ 0.05 ‘.’ 0.1 ‘ ’ 1
>   AIC(gn, g1, g2p, g2)
          df        AIC
gn   2.00000  -97.33578
g1   3.00000  -95.46085
g2p 34.23614 -419.46634
g2  33.27020 -420.70727
>   summary(g2)

Family: gaussian 
Link function: identity 

Formula:
response ~ scaled_date_por + s(scaled_date_doy, k = 5) + s(yearF, 
    bs = "re")

Parametric coefficients:
                Estimate Std. Error t value Pr(>|t|)    
(Intercept)     0.308195   0.008689   35.47   <2e-16 ***
scaled_date_por 0.004979   0.009058    0.55    0.583    
---
Signif. codes:  0 ‘***’ 0.001 ‘**’ 0.01 ‘*’ 0.05 ‘.’ 0.1 ‘ ’ 1

Approximate significance of smooth terms:
                      edf Ref.df       F p-value    
s(scaled_date_doy)  3.523   3.87 142.964 < 2e-16 ***
s(yearF)           26.747  70.00   0.625 0.00257 ** 
---
Signif. codes:  0 ‘***’ 0.001 ‘**’ 0.01 ‘*’ 0.05 ‘.’ 0.1 ‘ ’ 1

R-sq.(adj) =  0.631   Deviance explained = 66.4%
GCV = 0.017897  Scale est. = 0.016266  n = 354
```

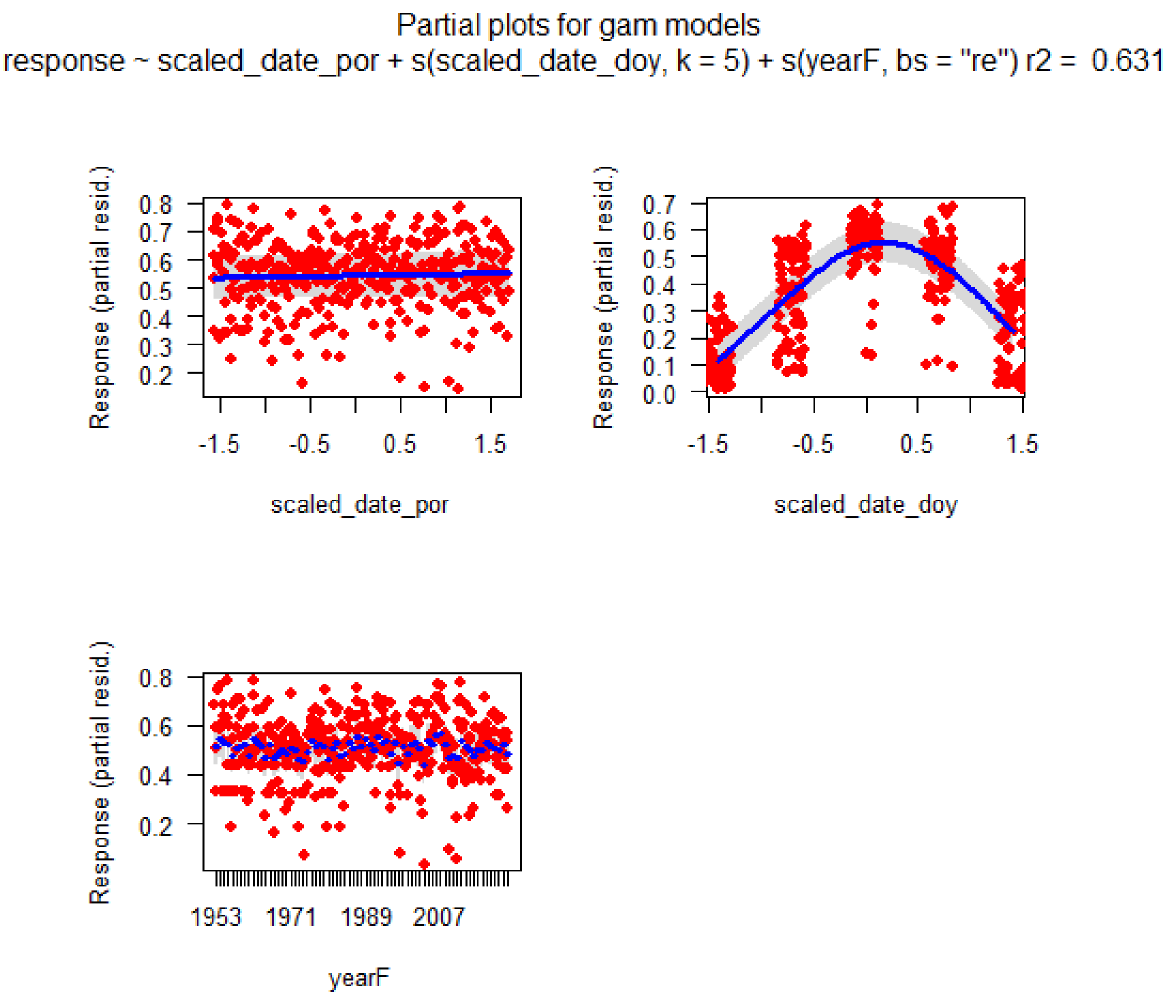

Figure S.9. (A) Model selection and General Additive Model results for proportion of growing season monthly flow in the Colorado River versus the Grand River Ditch; (B) partial residual plots for proportion of growing season monthly flow in the Colorado River versus the Grand River Ditch.

Ponded water

Figure S.10 provides visuals for ponded water results. Figures S.11 and S.12 give R output from GAM models of ponded water.

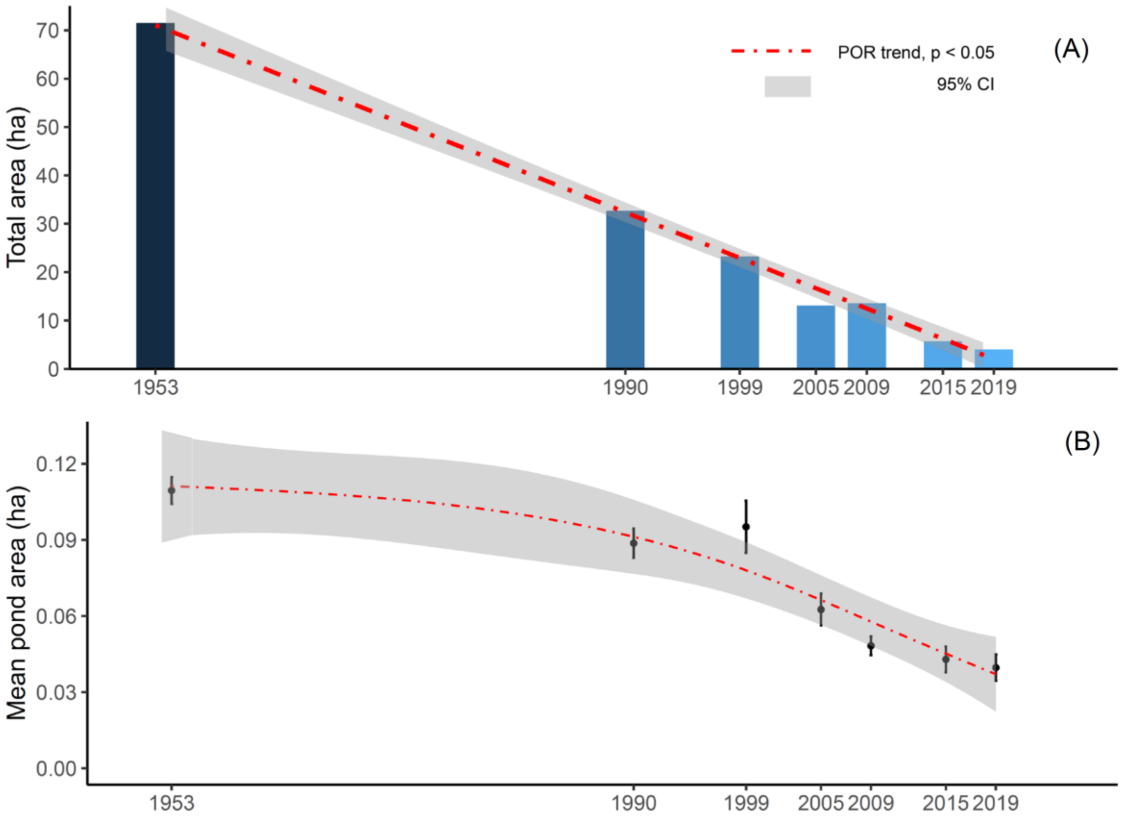

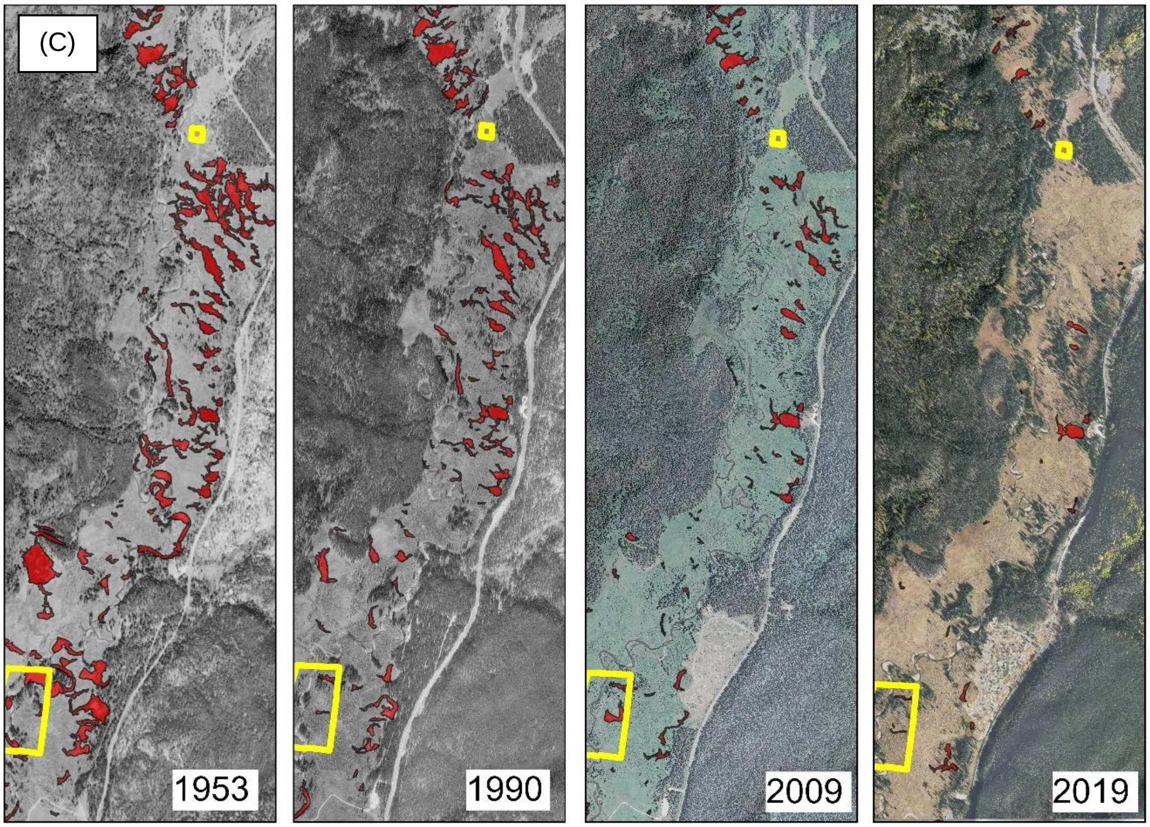

Figure S.10. Ponded water, 1953 – 2019, in the Kawuneeche Valley, Rocky Mountain National Park: (A) total pond area (ha) summarized across variable numbers of ponds delineated in each year; (B) mean pond size (ha). Legend in (A) applies to both panels. Error bands in (A) are a 95% confidence interval. Error bars in (B) are the standard errors around the mean pond area in each year. (C) Aerial images of example open water (primarily beaver ponds) across four years. Red polygons show beaver ponds digitized from growing season (7/9 to 10/23) imagery in each year. The region shown includes the primary sampled region (see Figure 2, main narrative) and northward up valley ~2 km. The Holzwarth (bottom of images) and Colorado River trailhead (top of images) exclosure boundaries are shown in yellow.

```
>     anova(gn, g1, g1b)
Analysis of Deviance Table

Model 1: sum_park_pond ~ 1
Model 2: sum_park_pond ~ scaled_date_por
Model 3: sum_park_pond ~ s(scaled_date_por, k = 5)
  Resid. Df Resid. Dev                Df Deviance      F         Pr(>F)
1         6       3285
2         5         19 1.0000000000000     3267 881.52 0.00000081276 ***
3         5         19 0.0000000000168        0   0.54 0.00000000022 ***
---
Signif. codes:  0 ‘***’ 0.001 ‘**’ 0.01 ‘*’ 0.05 ‘.’ 0.1 ‘ ’ 1
>     AIC(gn, g1, g1b)
    df  AIC
gn   2 66.9
g1   3 32.7
g1b  3 32.7
>     summary(g1)

Family: gaussian
Link function: identity

Formula:
sum_park_pond ~ scaled_date_por

Parametric coefficients:
                Estimate Std. Error t value   Pr(>|t|)
(Intercept)      23.4038     0.7276    32.2 0.00000055 ***
scaled_date_por  -1.0456     0.0352   -29.7 0.00000081 ***
---
Signif. codes:  0 ‘***’ 0.001 ‘**’ 0.01 ‘*’ 0.05 ‘.’ 0.1 ‘ ’ 1


R-sq.(adj) =  0.993   Deviance explained = 99.4%
GCV = 5.1878  Scale est. = 3.7056    n = 7
```

Figure S.11. Model selection and General Additive Model results for total pond area by year.

```
>           AIC(gn, g1, g1b)
      df    AIC
gn   2.00 -28.3
g1   3.00 -34.6
g1b 3.99 -42.8
>           summary(g1b)

Family: gaussian
Link function: identity

Formula:
mean_park_pond ~ s(scFullDate, k = 2)

Parametric coefficients:
            Estimate Std. Error t value Pr(>|t|)
(Intercept)  0.07009    0.00318    22.1 0.000021 ***
---
Signif. codes:  0 ‘***’ 0.001 ‘**’ 0.01 ‘*’ 0.05 ‘.’ 0.1 ‘ ’ 1

Approximate significance of smooth terms:
               edf Ref.df     F p-value
s(scFullDate) 1.93   1.99 25.1   0.005 **
---
Signif. codes:  0 ‘***’ 0.001 ‘**’ 0.01 ‘*’ 0.05 ‘.’ 0.1 ‘ ’ 1

R-sq.(adj) =  0.896   Deviance explained = 92.9%
-REML = -13.556  Scale est. = 7.0565e-05  n = 7
```

Figure S.12. Model selection and General Additive Model results for mean pond size by year.

Appendix S5: Ground water results

Table S.3 provides summary statistics and autocorrelation visuals for the ground water model. Ground water model results may be influenced by autocorrelation and should be used with some caution. Figure S.13 provides R output from GAM models of DTW.

Table S.3. (A) Confidence intervals and autocorrelation results for period of record ground water trend models.

Model	POR trend coefficient lower 95% CI	POR trend coefficient upper 95% CI	Lung Box autocorrelation test (chi-square statistic, approx.. degrees of freedom and p-value)	Autocorrelation (A/PACF) visual interpretation
Growing season DTW (cm)	Nonlinear, NA	Nonlinear, NA	X-squared = 478, df = 31, p-value <2e-16	Evidence of lags

```
Analysis of Deviance Table

Model 1: mean_dtw ~ 1
Model 2: mean_dtw ~ s(scaled_date_por) + s(scaled_date_doy, k = 5) + s(yearF,
    bs = "re")
Model 3: mean_dtw ~ s(scaled_date_por) + s(scaled_date_doy, k = 5) + s(yearF,
    bs = "re") + s(SiteName, bs = "re")
Model 4: mean_dtw ~ s(scaled_date_por) + s(scaled_date_doy, k = 5) + s(yearF,
    bs = "re") + s(SiteName, bs = "re") + s(D_2) + s(Q)
Model 5: mean_dtw ~ s(scaled_date_por) + s(scaled_date_doy, k = 5) + s(yearF,
    bs = "re") + s(SiteName, bs = "re") + D_2 + s(Q) + beav
Model 6: mean_dtw ~ s(scaled_date_por) + s(scaled_date_doy, k = 5) + s(yearF,
    bs = "re") + s(SiteName, bs = "re") + D_2 + s(Q) + beav +
    s(SWElagged)
  Resid. Df Resid. Dev     Df Deviance     F Pr(>F)
1       941    1384639
2       915     834611 26.417   550028  84.4 <2e-16 ***
3       900     321484 14.453   513128 143.9 <2e-16 ***
4       904     296062 -3.504    25422
5       900     223424  3.584    72638  82.2 <2e-16 ***
6       900     222719  0.381      705   7.5  0.027 *
---
Signif. codes:  0 ‘***’ 0.001 ‘**’ 0.01 ‘*’ 0.05 ‘.’ 0.1 ‘ ’ 1
>     AIC(gn, g1a, g1b, g1c, g1d, g1dx)
       df  AIC
gn    2.0 9547
g1a  27.0 9120
g1b  42.1 8252
g1c  36.3 8163
g1d  39.3 7904
g1dx 40.1 7902
> summary(g2)

Family: gaussian
Link function: identity

Formula:
mean_dtw ~ s(scaled_date_por) + s(scaled_date_doy, k = 5) + s(yearF,
    bs = "re") + s(SiteName, bs = "re") + D_2 + s(Q) + beav

Parametric coefficients:
            Estimate Std. Error t value   Pr(>|t|)
(Intercept)   -54.53      10.36   -5.26 0.00000018 ***
D_2            -8.89       2.11   -4.22 0.00002703 ***
beav1          47.62       2.69   17.72    < 2e-16 ***
---
Signif. codes:  0 ‘***’ 0.001 ‘**’ 0.01 ‘*’ 0.05 ‘.’ 0.1 ‘ ’ 1

Approximate significance of smooth terms:
                     edf Ref.df      F p-value
s(scaled_date_por)  3.52   3.65   5.63 0.00047 ***
s(scaled_date_doy)  1.94   2.40   5.97 0.00139 **
s(yearF)           13.99  19.00   4.65 < 2e-16 ***
s(SiteName)         8.96   9.00 207.86 < 2e-16 ***
s(Q)                6.89   7.94  23.45 < 2e-16 ***
---
Signif. codes:  0 ‘***’ 0.001 ‘**’ 0.01 ‘*’ 0.05 ‘.’ 0.1 ‘ ’ 1

R-sq.(adj) =  0.832   Deviance explained = 83.9%
GCV = 257.71  Scale est. = 247.23    n = 942
```

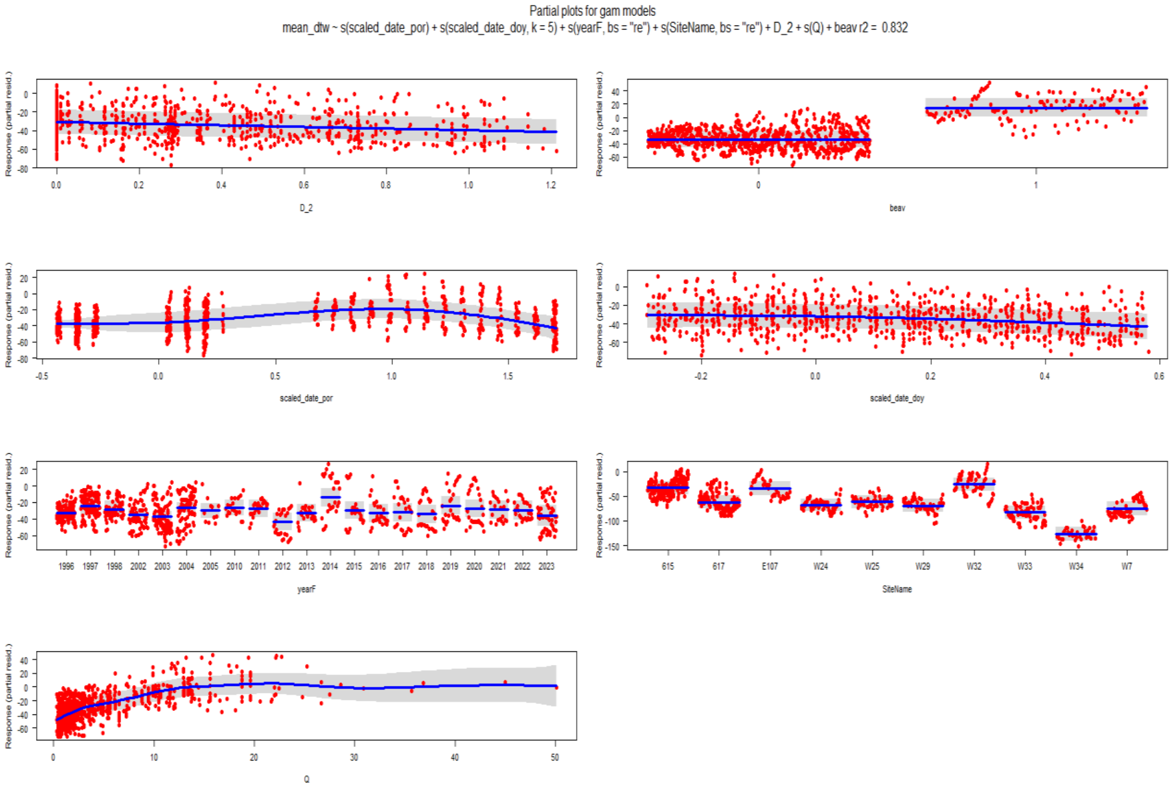

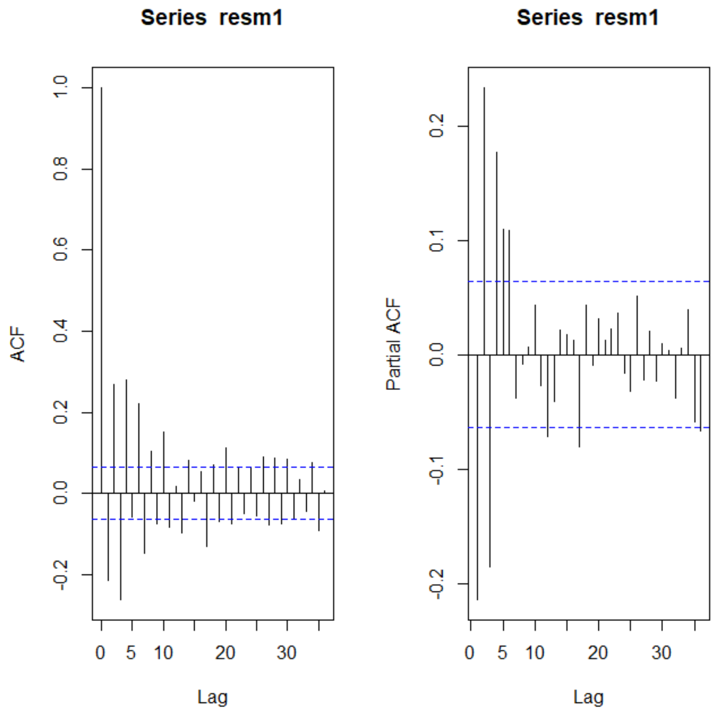

Figure S.13. (A) Model selection and General Additive Model for growing season weekly DTW; (B) partial residual plots for final model of growing season weekly DTW; (C) autocorrelation and partial autocorrelation plots for final model of growing season weekly DTW.

Appendix S6: Moose and elk results

The KV comprises a small area within Colorado Parks and Wildlife's Data Analysis Unit (DAU) E-8, within which elk population size and demographic information is estimated for the Troublesome elk herd (Colorado Parks and Wildlife 2024a, Oldham 2010, National Park Service 2007). Prior to 2010, the population objective for the Troublesome herd was 2,700 animals. From 1991 – 2009, post hunt population estimates regularly exceeded this objective, with a high estimate of 6,600 elk in 1995 (Oldham 2010). The population objective was adjusted to a range of 3,600 – 4,300 elk. In 2024, CPW released updated DRAFT Northwest Colorado Elk Herd Management Plans for several west slope elk DAUs including E-8. CPW proposed a preferred management alternative of 3,400 – 4,400 elk, which would expand the objective range slightly to allow for more management flexibility. Modeled post-harvest population estimates have been adjusted and describe the 1995 peak at nearly 6,000 animals (Table S.4; Colorado Parks and Wildlife 2024a). Regardless of which peak estimate is used, herd estimates have declined over time, with the 2023 post hunt population estimated at approximately 4,000 individuals (Table S.4; Colorado Parks and Wildlife 2024a). CPW's Data Analysis Unit DAU E-9 shares its western boundary with DAU E-8 and elk population size and demographic information are estimated for the E-9 St. Vrain herd (Colorado Parks and Wildlife 2024b, Huwer 2007). E-9 elk winter to the east of the Continental Divide, both in Rocky Mountain National Park, the town of Estes Park, CO and in the foothills of the Front Range, however a subset of these animals summer in the KV, mixing with a subset of animals from the E-8 herd. The population objective for the herd is 2,400 animals (with an acceptable range between 2,200 – 2,600 animals). The E-9 elk herd sat above objective from 1988 through the mid-2000s, peaking in 1999 at 4,400 animals, and have since declined to 2,130 animals in 2023 (Colorado Parks and Wildlife 2024b, Huwer 2007). The NPS conducts their own surveys and population estimates on the portion of the winter range that falls within eastern RMNP, and these data show even sharper declines from the peak in the early 2000s (Hobbs and Abouelezz 2020). While there is not specific information describing elk herbivory on willows in the KV, the highest DAU E-8 and E-9 population estimates coincide with earlier periods where willow abundance, height and cover were declining, however elk populations in both E-8 and the RMNP east side winter range have since declined (Colorado Parks and Wildlife 2024a, Colorado Parks and Wildlife 2024b, Hobbs and Abouelezz 2020, Huwer 2007), as moose densities on the landscape that includes the KV have increased (Abouelezz and Hobbs 2025, Dungan 2007). Future work is needed to estimate elk populations specific to the KV summer range, and to determine if the larger scale patterns in the E-8 and E-9 DAUs, and more local scale RMNP east side winter range estimates reflect dynamics on the west side of the park. Additional work may also be needed to understand moose densities relative to the KV valley floor, specifically, as moose densities presented in Abouelezz and Hobbs (2025) and Dungan (2007) describe densities on larger landscapes that include the KV.

Table S.4. Colorado Parks and Wildlife Data Analysis Unit (DAU) E-8 modeled total elk population size for the Troublesome elk herd as described in the DRAFT Northwest Colorado Elk Herd Management Plans (Colorado Parks and Wildlife 2024a).

Year	Total	Year	Total
1991	4779.01	2008	4223.13
1992	4970.92	2009	4744.83
1993	4712.41	2010	4125.29
1994	5148.88	2011	3920.06
1995	5901.36	2012	4408.90
1996	5578.60	2013	4438.84
1997	4970.71	2014	4590.58
1998	4764.11	2015	4746.82
1999	4719.99	2016	4719.58
2000	4739.23	2017	4974.15
2001	5269.71	2018	4480.28
2002	5073.54	2019	4336.93
2003	5050.04	2020	4166.90
2004	4776.77	2021	4231.28
2005	4524.30	2022	4063.55
2006	4289.28	2023	4029.32
2007	4183.16		

Appendix S7: Vegetation results

Willow stem height and patch area

Figure S.14 shows R output for GAM models of willow stem height in and outside three exclosures. Figure S.15 provides examples of willow patch area digitized from aerial imagery. Figure S.16 gives visuals of willow patch size and total area results. Figures S.17 and S.18 give R output for models of willow patch size and total area.

```
> anova(nullgam, gm)
Analysis of Deviance Table

Model 1: plant_ht_cm ~ 1
Model 2: plant_ht_cm ~ s(scFullDate, k = 5, by = FencedF) + FencedF
  Resid. Df Resid. Dev   Df Deviance    F Pr(>F)
1       362    4581463
2       357    1950813 5.37  2630650 89.8 <2e-16 ***
---
Signif. codes:  0 ‘***’ 0.001 ‘**’ 0.01 ‘*’ 0.05 ‘.’ 0.1 ‘ ’ 1
>       summary(gm)

Family: gaussian
Link function: identity

Formula:
plant_ht_cm ~ s(scFullDate, k = 5, by = FencedF) + FencedF

Parametric coefficients:
                Estimate Std. Error t value Pr(>|t|)
(Intercept)       246.47       5.86    42.1   <2e-16 ***
FencedFUnfenced  -156.48       7.86   -19.9   <2e-16 ***
---
Signif. codes:  0 ‘***’ 0.001 ‘**’ 0.01 ‘*’ 0.05 ‘.’ 0.1 ‘ ’ 1

Approximate significance of smooth terms:
                              edf Ref.df    F   p-value
s(scFullDate):FencedFFenced   1.00   1.00 24.3 0.0000018 ***
s(scFullDate):FencedFUnfenced 2.59   2.97 21.3   < 2e-16 ***
---
Signif. codes:  0 ‘***’ 0.001 ‘**’ 0.01 ‘*’ 0.05 ‘.’ 0.1 ‘ ’ 1

R-sq.(adj) =  0.569   Deviance explained = 57.4%
-REML = 2066.2  Scale est. = 5458.2    n = 363
```

```
Analysis of Deviance Table

Model 1: plant_ht_cm ~ 1
Model 2: plant_ht_cm ~ s(scFullDate, k = 5, by = FencedF) + FencedF
  Resid. Df Resid. Dev   Df Deviance   F Pr(>F)
1      1166    4928445
2      1157    1977311 9.14  2951134 189 <2e-16 ***
---
Signif. codes:  0 ‘***’ 0.001 ‘**’ 0.01 ‘*’ 0.05 ‘.’ 0.1 ‘ ’ 1
>       summary(gm)

Family: gaussian
Link function: identity

Formula:
plant_ht_cm ~ s(scFullDate, k = 5, by = FencedF) + FencedF

Parametric coefficients:
                Estimate Std. Error t value Pr(>|t|)
(Intercept)       109.84       1.92    57.2   <2e-16 ***
FencedFUnfenced   -59.81       2.49   -24.1   <2e-16 ***
---
Signif. codes:  0 ‘***’ 0.001 ‘**’ 0.01 ‘*’ 0.05 ‘.’ 0.1 ‘ ’ 1

Approximate significance of smooth terms:
                               edf Ref.df   F p-value
s(scFullDate):FencedFFenced   3.74   3.95 221  <2e-16 ***
s(scFullDate):FencedFUnfenced 3.41   3.76  19  <2e-16 ***
---
Signif. codes:  0 ‘***’ 0.001 ‘**’ 0.01 ‘*’ 0.05 ‘.’ 0.1 ‘ ’ 1

R-sq.(adj) =  0.596   Deviance explained = 59.9%
-REML = 6000.3  Scale est. = 1707.8    n = 1167
>
```

```
Analysis of Deviance Table

Model 1: plant_ht_cm ~ 1
Model 2: plant_ht_cm ~ s(scFullDate, k = 5, by = FencedF) + FencedF
  Resid. Df Resid. Dev   Df Deviance   F Pr(>F)
1       762    2989188
2       753     957647 9.06  2031541 177 <2e-16 ***
---
Signif. codes:  0 ‘***’ 0.001 ‘**’ 0.01 ‘*’ 0.05 ‘.’ 0.1 ‘ ’ 1
>       summary(gm)

Family: gaussian
Link function: identity

Formula:
plant_ht_cm ~ s(scFullDate, k = 5, by = FencedF) + FencedF

Parametric coefficients:
                Estimate Std. Error t value Pr(>|t|)
(Intercept)       112.38       1.89    59.5   <2e-16 ***
FencedFUnfenced   -47.95       2.65   -18.1   <2e-16 ***
---
Signif. codes:  0 ‘***’ 0.001 ‘**’ 0.01 ‘*’ 0.05 ‘.’ 0.1 ‘ ’ 1

Approximate significance of smooth terms:
                              edf Ref.df     F  p-value
s(scFullDate):FencedFFenced   3.48   3.81 345.4  < 2e-16 ***
s(scFullDate):FencedFUnfenced 3.35   3.71   9.3 0.000006 ***
---
Signif. codes:  0 ‘***’ 0.001 ‘**’ 0.01 ‘*’ 0.05 ‘.’ 0.1 ‘ ’ 1

R-sq.(adj) =  0.676   Deviance explained =   68%
-REML = 3808.6  Scale est. = 1269.8    n = 763
```

Figure S.14. Model selection and General Additive Model for willow stem height in three exclosures: (A) Colorado River Trailhead; (B) Holzwarth; (C) Gaskill.

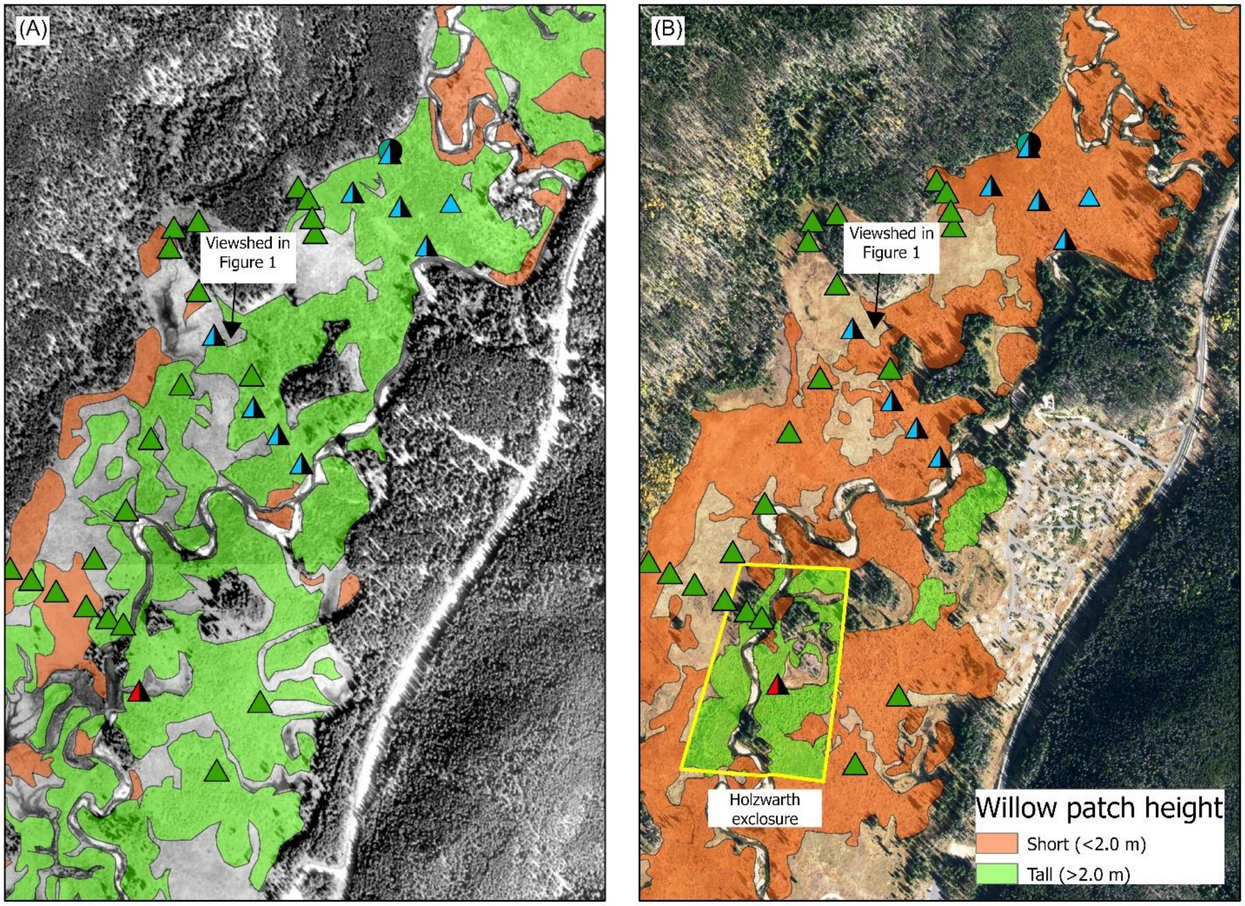

Figure S.15. Example of tall (>2.0 m; lime green) and short (orange) willow patches in the Kawuneeche valley, Rocky Mountain National Park: (A) 1999 and (B) 2019. Polygons were hand digitized from DOQQ imagery in 1999 and NAIP imagery in 2019. Legend in (B) applies to both panels. Holzwarth exclosure in (B) was constructed in 2011. A subset of sample locations is shown in both panels with colored symbols as in Figure 2 in the main paper. The viewshed for Figure 1 in the main paper is shown with label and arrows.

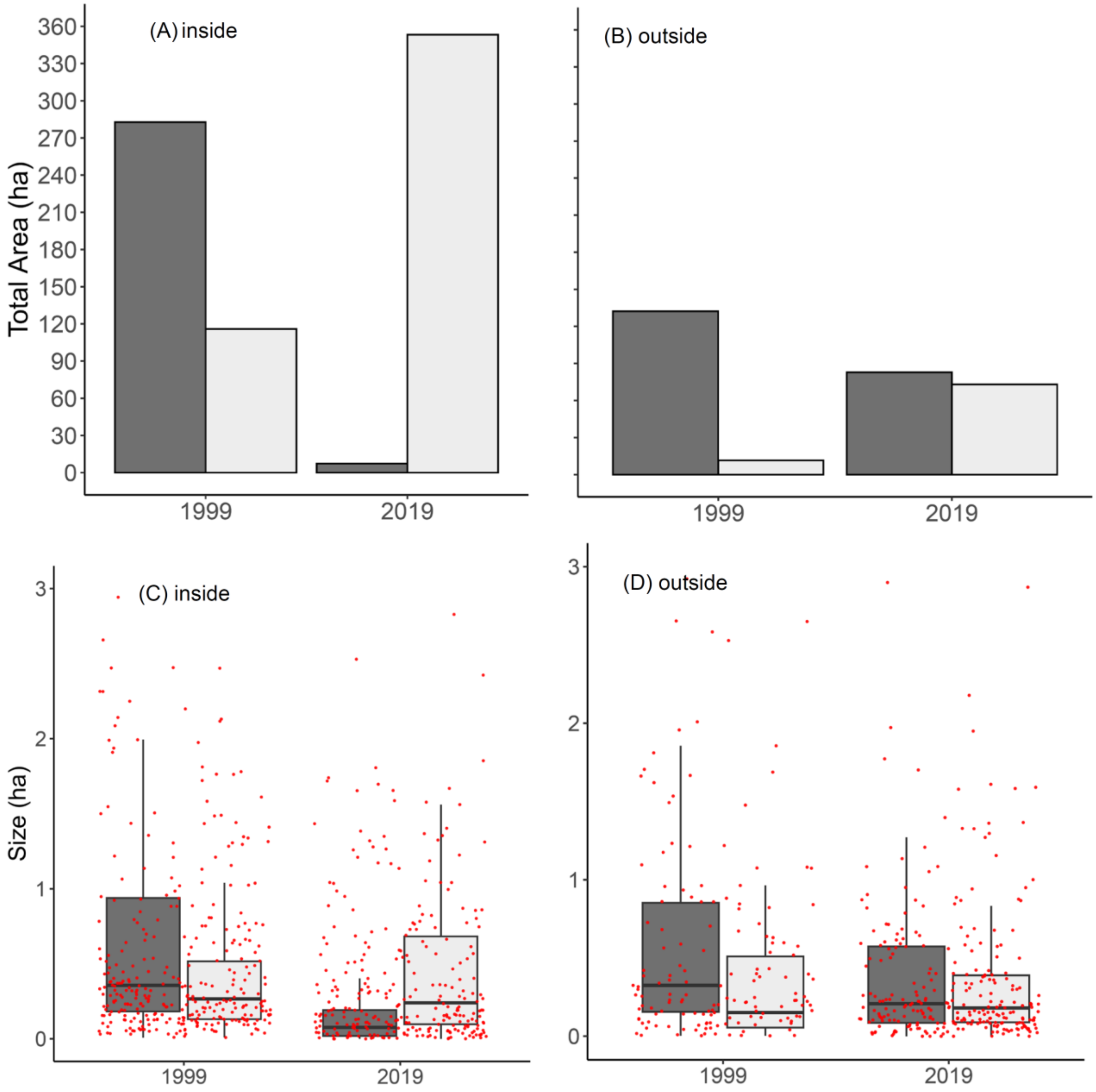

Figure S.16. Area and patch size of tall (dark gray bars, >= 2.0 m) and short (light gray bars, < 2.0 m) willow in the Kawuneeche Valley, 1999 and 2019: (A) total area inside Rocky Mountain National Park; (B) total area outside the park; (C) median patch size inside the park; (D) patch size outside the park. Willow area within exclosures is omitted in all comparisons.

```
>  #Tall willow patch in the park
>     z

        Kruskal-Wallis rank sum test

data:  Area_ha by YearF
Kruskal-Wallis chi-squared = 58, df = 1, p-value = 2e-14

> #Short willow patch in the park
>     a

        Kruskal-Wallis rank sum test

data:  Area_ha by YearF
Kruskal-Wallis chi-squared = 0.3, df = 1, p-value = 0.6

>  #Tall willow patch outside the park
>     x

        Kruskal-Wallis rank sum test

data:  Area_ha by YearF
Kruskal-Wallis chi-squared = 11, df = 1, p-value = 0.0008

>  #Short willow patch outside the park
>     y

        Kruskal-Wallis rank sum test

data:  Area_ha by YearF
Kruskal-Wallis chi-squared = 1, df = 1, p-value = 0.3
```

Figure S.17. Kruskal-Wallis models of mean willow patch size by size inside and outside Rocky Mountain National Park

```
>     #Tall willow patch inside the park
>     cat("Observed difference in sums:", observed_diff, "\n")
Observed difference in sums: 275
>     cat("Permuted difference in sums:", perm_diff, "\n")
Permuted difference in sums: 156
>     cat("p-value:", p_value, "\n")
p-value: 0
>     #Short willow patch inside the park
>     cat("Observed difference in sums:", observed_diff, "\n")
Observed difference in sums: -237
>     cat("Permuted difference in sums:", perm_diff, "\n")
Permuted difference in sums: -110
>     cat("p-value:", p_value, "\n")
p-value: 0.0113
>     #Tall willow patch outside the park
>     cat("Observed difference in sums:", observed_diff, "\n")
Observed difference in sums: 49.4
>     cat("Permuted difference in sums:", perm_diff, "\n")
Permuted difference in sums: -37.7
>     cat("p-value:", p_value, "\n")
p-value: 0.228
>     #Short willow patch outside the park
>     cat("Observed difference in sums:", observed_diff, "\n")
Observed difference in sums: 61.5
>     cat("Permuted difference in sums:", perm_diff, "\n")
Permuted difference in sums: 63.2
>     cat("p-value:", p_value, "\n")
p-value: 0.233
```

Figure S.18. Permutation models of total willow area by size inside and outside Rocky Mountain National Park.

Vegetation composition

We provide R output in Figures S.19 to S.21 from the ordination of 1998 and 2021 vegetation communities. Table S.5 gives the mean absolute cover of plant species across community type and year.

```
> nd

Call:
metaMDS(comm = veg98, distance = "bray", autotransform = FALSE)

global Multidimensional Scaling using monoMDS

Data:     veg98
Distance: bray

Dimensions: 2
Stress:     0.1792564
Stress type 1, weak ties
Best solution was repeated 1 time in 20 tries
The best solution was from try 13 (random start)
Scaling: centring, PC rotation, halfchange scaling
Species: expanded scores based on 'veg98'
```

```
> summary(ano)

Call:
anosim(x = v.dist, grouping = data.scorest$WetType, permutations = 10000)
Dissimilarity: bray

ANOSIM statistic R: 0.608
      Significance: 9.999e-05

Permutation: free
Number of permutations: 10000
```

Figure S.19. Ordination model of vegetation composition in 1998.

```
> nd21

Call:
metaMDS(comm = veg21, distance = "bray", autotransform = FALSE)

global Multidimensional Scaling using monoMDS

Data:     veg21
Distance: bray

Dimensions: 2
Stress:     0.1990758
Stress type 1, weak ties
Best solution was not repeated after 20 tries
The best solution was from try 3 (random start)
Scaling: centring, PC rotation, halfchange scaling
Species: expanded scores based on 'veg21'
```

```
> ano21

Call:
anosim(x = v.dist21, grouping = data.scorest2$WetType, permutations = 10000)
Dissimilarity: bray

ANOSIM statistic R: 0.5636
      Significance: 9.999e-05

Permutation: free
Number of permutations: 10000
```

Figure S.20. Ordination model of vegetation composition in 2021.

```
> env98

***VECTORS

                              NMDS1    NMDS2     r2 Pr(>r)
n.Abs.sumcov.inv           -0.29258  0.95624 0.3205  0.004 **
c.C.Mean.all                0.38920 -0.92116 0.7449  0.001 ***
ws.WetAff.Mean.Score.All    0.61300 -0.79008 0.5453  0.001 ***
bp_99_proportion            0.48540  0.87429 0.0784  0.307
wil_99_Short_proportion     0.27951  0.96014 0.0565  0.467
wil_99_Tall_proportion     -0.62603  0.77980 0.3107  0.006 **
---
Signif. codes:  0 ‘***’ 0.001 ‘**’ 0.01 ‘*’ 0.05 ‘.’ 0.1 ‘ ’ 1
Permutation: free
Number of permutations: 999
```

```
> env21

***VECTORS

                              NMDS1    NMDS2     r2 Pr(>r)
n.Abs.sumcov.inv           -0.99011 -0.14031 0.6885  0.001 ***
c.C.Mean.all                0.99692  0.07838 0.6757  0.001 ***
ws.WetAff.Mean.Score.All    0.95643 -0.29196 0.7805  0.001 ***
bp_19_proportion            0.12251  0.99247 0.0822  0.339
wil_19_Short_proportion    -0.94882 -0.31582 0.2086  0.042 *
wil_19_Tall_proportion     -0.47294  0.88109 0.2312  0.013 *
---
Signif. codes:  0 ‘***’ 0.001 ‘**’ 0.01 ‘*’ 0.05 ‘.’ 0.1 ‘ ’ 1
Permutation: free
Number of permutations: 999
```

Figure S.21. Vegetation metric relationships to vegetation composition in 1998 and 2021.

Table S.5. Mean absolute cover of 28 key plant species across community type and year in the Kawuneeche Valley, Rocky Mountain National Park. The Table is sorted from most to least abundant taxa. Taxa included are all indicator species and any other taxa with a mean absolute cover across year and wetland type >= 3.0.

	Beaver pond		Fen		Riparian willow		Grass land	
**Species**	**1998**	**2021**	**1998**	**2021**	**1998**	**2021**	**2021**	**Mean cover**
Carex utriculata	69.67	54.17	34.83	25.67	8.21	35.17	11.79	34.21
Salix planifolia	87.50	37.50	32.63	22.94	6.38	5.50	4.50	28.13
Calamagrostis canadensis	5.50	9.50	18.00	36.33	52.00	54.17	6.78	26.04
Carex aquatilis	7.83	40	37.28	36.72	13.50	17.50	11.72	23.51
Deschampsia cespitosa	29.21	2.80	19.70	3.83	14.30	3.00	8.17	11.57
Salix geyeriana	0.50	0.50	11.50	5.50	36.58	5.50	7.00	9.58
Trifolium repens	21.50	17.50	0.50	0	18.06	0	7.34	9.27
Fragaria virginiana	0	37.50	0.50	3.00	8.94	0	14.30	9.18
Carex microptera	5.50	0	37.50	5.50	3.83	0	9.38	8.82
Danthonia intermedia	11.50	0	0	0	5.50	0	37.50	7.79
Pinus contorta var. latifolia	0	0	4.90	14.50	11.50	0	20.17	7.30
Carex canescens	9.00	9.00	21.50	0	5.50	5.50	0	7.21
Phalaris arundinacea	0	0	0	0	0	0	46.50	6.64
Polemonium occidentale ssp. Occidentale	7.83	5.50	7.25	5.50	6.67	5.50	3.00	5.89
Phleum alpinum	11.50	0	11.50	0	11.50	0	5.72	5.75
Poa pratensis	0.10	3.00	0	5.50	3.00	0	26.08	5.38
Salix wolfii	0	0	17.07	3.83	8.50	0	5.50	4.99
Salix monticola	5.50	5.50	8.50	5.50	8.39	0	1.27	4.95
Potentilla fruticosa	4.25	0.50	5.83	8.50	5.50	0	7.50	4.58
Pedicularis groenlandica	5.50	5.50	7.83	3.83	0.50	0	5.50	4.10
Psychrophila leptosepala	5.50	0.50	13.50	5.50	0	0	0	3.57
Betula glandulosa	0	0	16.36	6.90	0	0	0	3.32
Eriophorum angustifolium	17.50	5.50	0	0	0	0	0	3.29
Clementsia rhodantha	5.50	5.50	8.50	3.00	0	0	0	3.21
Picea engelmannii	0	0	0.30	17.50	0.50	0	3.83	3.16
Taraxacum officinale	3.00	5.50	0	0	2.38	0.50	5.12	2.36
Tragopogon pratensis	5.50	0	0	0	5.50	0	0.30	1.61
Glyceria striata	0.50	0	0	1.75	0	0	0	0.32

Appendix S8: Historical photographs

Figures S.22 and S.23 provide historical photos of wetland areas in the Kawuneeche Valley. The images show extensive cover of tall willow.

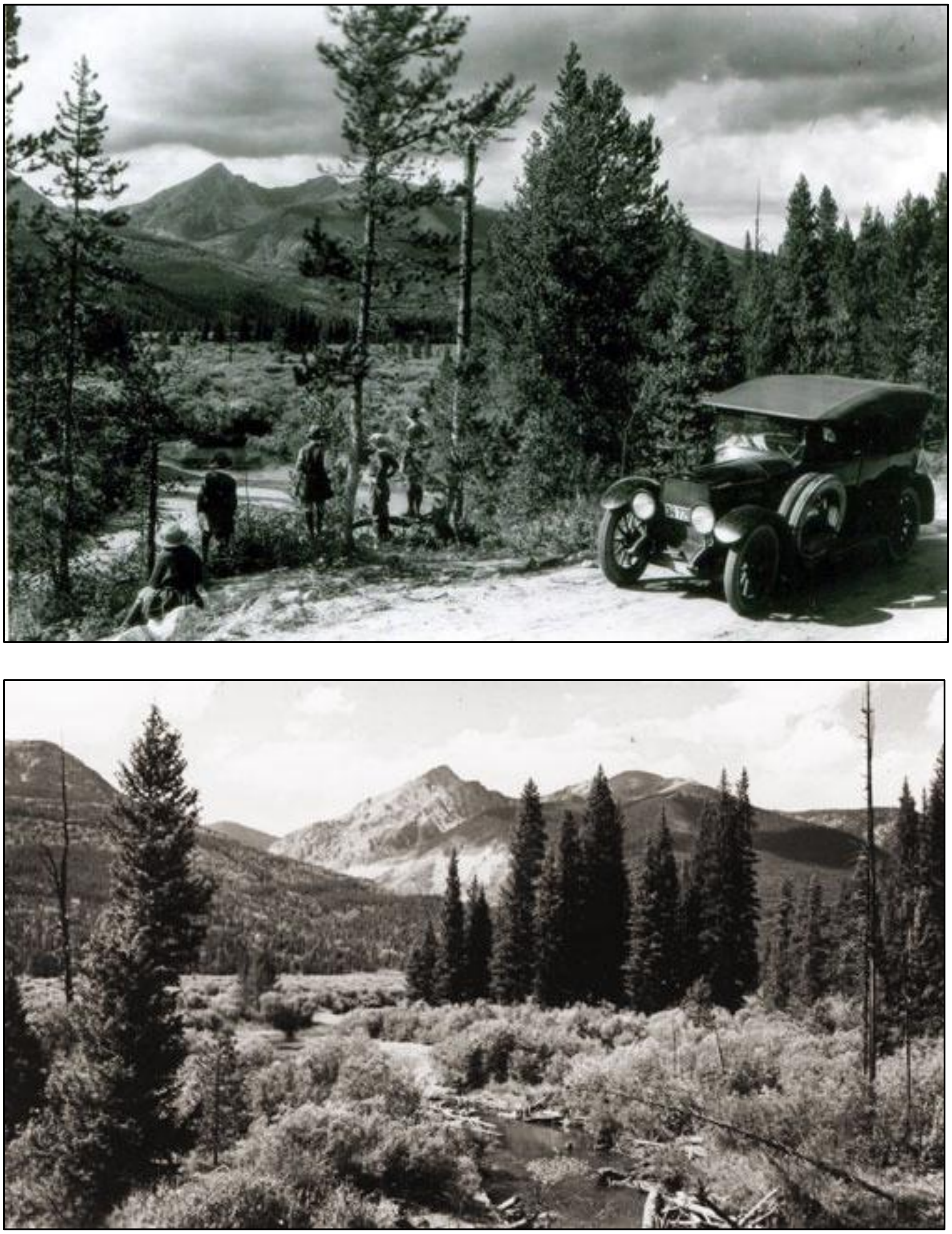

Figure S.22. Two photos taken in 1922 of wetlands in the central portion of Kawuneeche Valley, Rocky Mountain National Park looking west toward Baker Peak. Images show extensive tall riparian willow stands.

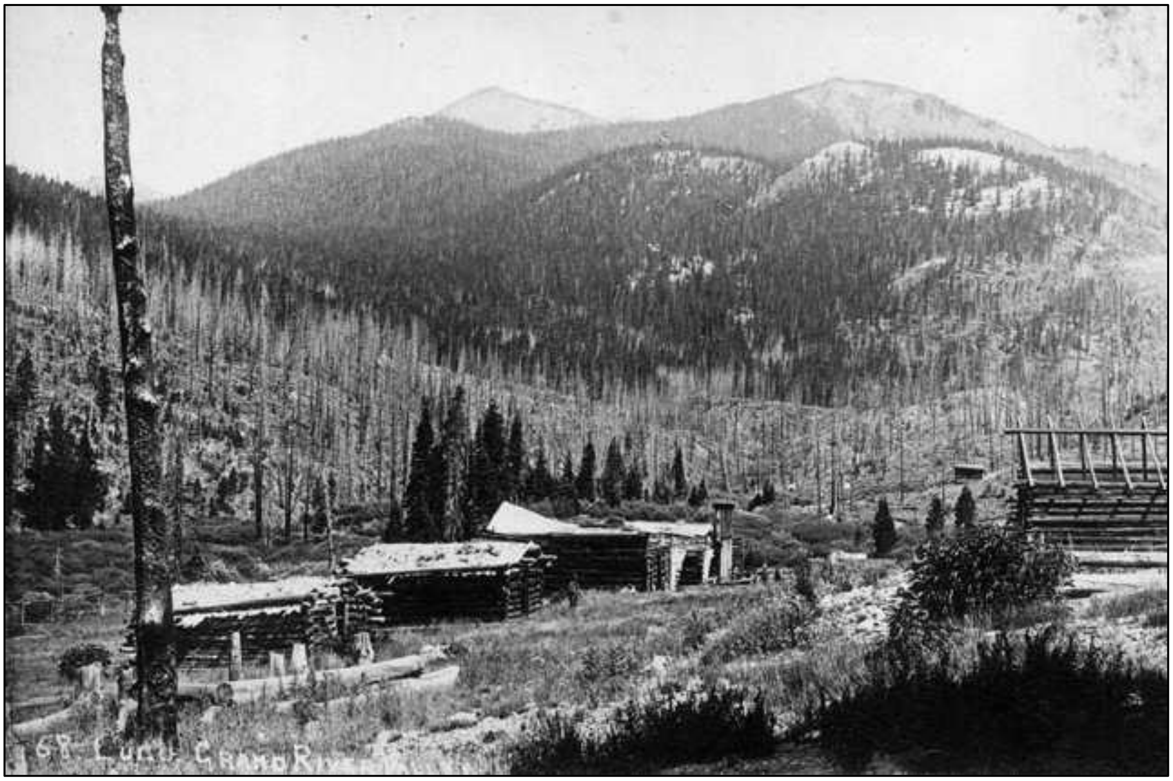

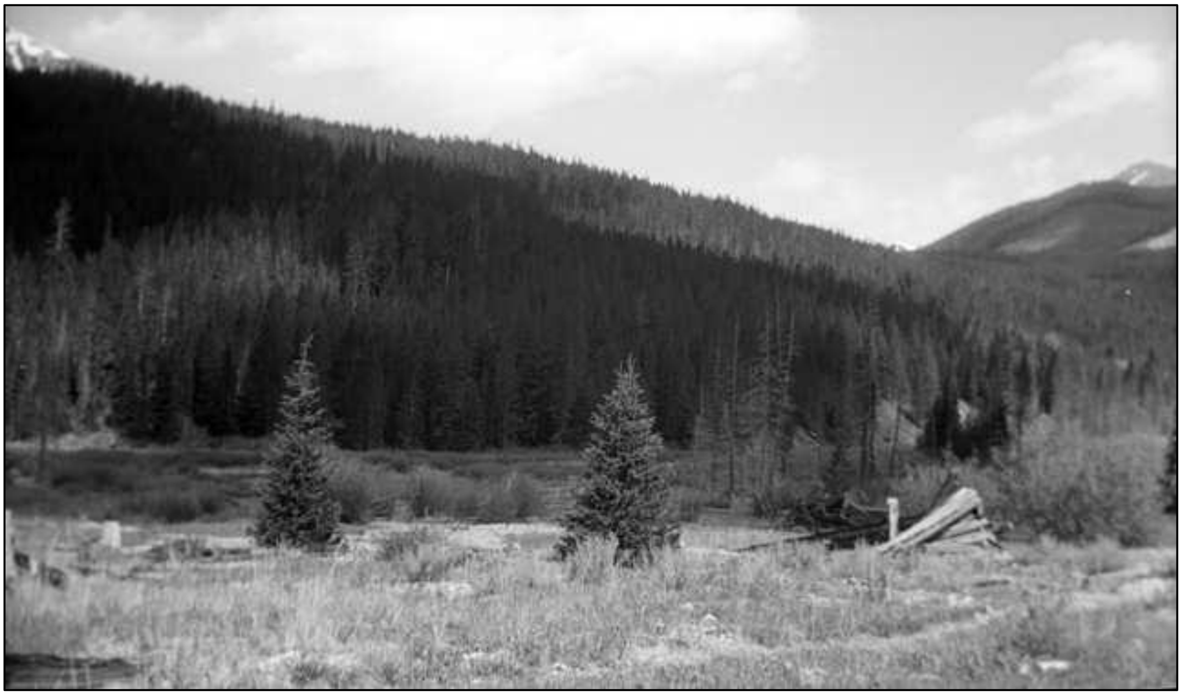

Figure S.23. Top photo from July 1889, and lower photo from 1950s, of the Lulu City townsite in the northern portion of the Kawuneeche Valley, Rocky Mountain National Park. Tall willows are abundant along the Colorado River just to the left of the cabins (and cabin remnants).

Literature Cited

Abouelezz, H.G. and Hobbs, N.T. (2025). A high-altitude thermal infrared method for estimating moose abundance and demography in Rocky Mountain National Park, USA. Wildlife Biology. e01368. https://doi.org/10.1002/wlb3.01368

Abatzoglou J. T. (2012). Development of gridded surface meteorological data for ecological applications and modelling. International Journal of Climatology. doi: https://doi.org/10.1002/joc.3413

Braddock, W.A. and Cole, J.C. (1990). Geologic map of Rocky Mountain National Park and vicinity, Colorado (No. 1973).

Byers, J. E., S. Reichard, J. M. Randall, I. M. Parker, C. S. Smith, W. M. Lonsdale, I. A. E. Atkinson, T. R. Seastedt, M. Williamson, E. Chornesky, and D. Hayes. (2002). Directing research to reduce the impacts of nonindigenous species. Conservation Biology 16:630–640.

Carlson, A. R., Sibold, J. S., and Negrón, J. F. (2021). Wildfire and spruce beetle outbreak have mixed effects on below-canopy temperatures in a Rocky Mountain subalpine forest. Journal of Biogeography, 48, 216–230. https://doi.org/10.1111/jbi.13994

Carroll, R.W., Niswonger, R.G., Ulrich, C., Varadharajan, C., Siirila-Woodburn, E.R. and Williams, K.H. (2024). Declining groundwater storage expected to amplify mountain streamflow reductions in a warmer world. Nature Water, 2(5), pp.419-433.

Christian, J. M., and S. D. Wilson. (1999). Long-term ecosystem impacts of an introduced grass in the northern Great Plains. Ecology 80:2397–2407.

Colorado Parks and Wildlife. (2024a) Draft Northwest Colorado Elk Herd Management Plans: Troublesome elk herd management plan, data analysis unit E-08. https://cpw.state.co.us/thingstodo/Pages/HerdManagementPlans.aspx

Colorado Parks and Wildlife. (2024b). Elk 2023 post hunt population and sex ratio estimates. Population Estimates Reports. https://cpw.widen.net/s/hcvch7cgrc/2023-elk-population-estimates.

Cook, B. I., Ault, T. R., and Smerdon, J. E. (2015). Unprecedented 21st century drought risk in the American Southwest and Central Plains. Science Advances, 1, e1400082.

Croke, B. F., F. Andrews, A. J. Jakeman, S. Cuddy, and A. Luddy (2005). Redesign of the IHACRES rainfall-runoff model. 29th Hydrology and Water Resources Symposium, 21–23 February, 2005, Canberra, Australia.

Dennison, P. E., Brewer, S. C., Arnold, J. D., Moritz, M. A. (2014). Geophysical Research Letters. Geophysical Research Letters, 41, 2928–2933. https://doi.org/10.1002/2014GL059576

Dufrêne, M. and Legendre, P., (1997). Species assemblages and indicator species: the need for a flexible asymmetrical approach. Ecological monographs, 67(3), pp.345-366.

Ehrenfeld, J. G. (2003). Effects of exotic plant invasions on soil nutrient cycling processes. Ecosystems 6:503–523.

Environmental Systems Research Institute (ESRI) (2023). ArcGIS Pro Version 3.1. Redlands, CA: Environmental Systems Research Institute.

Fertig, W. (2011). Determining the nativity of plant species. Newsletter of the Utah Native Plant Society. https://www.unps.org/segolily/Sego2011SepOct.pdf. Accessed November 2020.

Frank, J. M., Massman, W. J., Ewers, B. E., Huckaby, L. S., Negron, J. F. (2014). Ecosystem CO2/H2O fluxes are explained by hydraulically limited gas exchange during tree mortality from spruce bark beetles. Journal of Geophysical Research: Biogeosciences, 119, 1195–1215. https://doi.org/10.1002/2013JG002597.Received

Frank, J. M., Massman, W. J., Ewers, B. E., Williams, D. G. (2019). Bayesian Analyses of 17 Winters of Water Vapor Fluxes Show Bark Beetles Reduce Sublimation Water Resources Research. Water Resources Research, 55, 1598–1623. https://doi.org/10.1029/2018WR023054

Fridley, J. D., J. J. Stachowicz, S. Naeem, D. F. Sax, E. W. Seabloom, M. D. Smith, T. J. Stohlgren, D. Tilman, and B. Von Holle. (2007). The invasion paradox: reconciling pattern and process in species invasions. Ecology 88:3–17.

Gangopadhyay, S., Woodhouse, C.A., McCabe, G.J., Routson, C.C. and Meko, D.M. (2022). Tree rings reveal unmatched 2nd century drought in the Colorado River Basin. Geophysical Research Letters, 49(11), p.e2022GL098781.

Gonzalez, P., Wang, F., Notaro, M., Vimont, D. J., and Williams, J. W. (2018). Disproportionate magnitude of climate change in United States national parks. Environmental Research Letters, 13, 104001.

Grace, J. B., and K. M. Irvine. (2020). Scientist's guide to developing explanatory statistical models using causal analysis principles. Ecology 101(4):p.e02962.

Grace, J. B., D. R. Schoolmaster Jr., J. Pearl, G. R. Guntenspergen, A. M. Little, B. R. Mitchell, K. M. Miller, and E. W. Schweiger. (2012). Guidelines for a graph-theoretic implementation of structural equation modeling. Ecosphere 3:73.

Hall, M., and Fagre, D. (2003). Modeled climate-inducted glacier changes in Glacier National Park. Bioscience 53, 131-140.

Harder, P., Pomeroy, J. W., and Westbrook, C. J. (2015). Hydrological resilience of a Canadian Rockies headwaters basin subject to changing climate, extreme weather, and forest management. Hydrological Processes, 29(18), 3905–3924. https://doi.org/10.1002/hyp.10596

Herman, K. D., L. A. Masters, M. R. Penskar, A. A. Reznicek, G. S. Wilhelm, and W. R. Brodowicz. (1997). Floristic quality assessment: development and application in the state of Michigan (USA). Natural Areas Journal 17:265–279.

Hobbs, N.T. and Abouelezz, H. (2020). Modeling in support of adaptive management of the Rocky Mountain National Park elk population, 2014-2019. Rocky Mountain National Park, Estes Park, Colorado. 45p. https://irma.nps.gov/DataStore/Reference/Profile/2275640

Hock, R. (2003). Temperature index melt modelling in mountain areas. Journal of hydrology, 282(1-4), 104-115.

Huwer, S. (2007). Elk management plan Data Analysis Unit E-9 St. Vrain Herd Game Management Unit 20. Colorado Division of Wildlife. Northeast Region, CO. 66p. https://cpw.widencollective.com/assets/share/asset/dh8akokgnr

Jennings, K. S., T. S. Winchell, B. Livneh, and N. P. Molotch. (2018). Spatial variation of the rain–snow temperature threshold across the Northern Hemisphere. Nature Communications 9(1):1148.

Karran, D. J., Westbrook, C. J., and Bedard-Haughn, A. (2018). Beaver-mediated water table dynamics in a Rocky Mountain fen. Ecohydrology, 11(2), e1923.

Koenker, R. (2023). quantreg: Quantile Regression. R package version 5.97, https://cran.r-project.org/web/packages/quantreg

Levine, J. M., M. Vilà, C. M. D'Antonio, J. S. Dukes, K. Grigulis, and S. Lavorel. (2003). Mechanisms underlying the impacts of exotic plant invasions. Proceedings of the Royal Society of London: Biological Sciences 270:775–781.

Ling, S. D., and Keane, J. P. (2024). Climate-driven invasion and incipient warnings of kelp ecosystem collapse. Nature Communications 15:1, 15(1), 1–9. https://doi.org/10.1038/s41467-023-44543-x

Ljung, G.M. and Box, G.E. (1978). On a measure of lack of fit in time series models. Biometrika, 65(2), 297-303.

Loheide, S. P., and Gorelick, S. M. (2007). Riparian hydroecology: A coupled model of the observed interactions between groundwater flow and meadow vegetation patterning. Water Resources Research, 43(7), 7414. https://doi.org/10.1029/2006WR005233

Lutz, J. A., van Wagtendonk, J. W., and Franklin, J. F. (2010). Climatic water deficit, tree species ranges, and climate change in Yosemite National Park. Journal of Biogeography, 37(5), 936–950. https://doi.org/10.1111/J.1365-2699.2009.02268.X

Matthews, J. W., G. Spyreas, and C. M. Long. (2015). A null model test of Floristic Quality Assessment: Are plant species' Coefficients of Conservatism valid? Ecological Indicators 52:1–7.

McCune, B., and Mefford, M.J. (2018). PC-ORD (7.07). MjM Software Design.

McKernan, C., Cooper, D. J., and Schweiger, E. W. (2018). Glacial loss and its effect on riparian vegetation of alpine streams. Freshwater Biology, 63(6), 518–529. https://doi.org/10.1111/FWB.13088

Meiners, S. J., S. A. Pickett, and M. L. Cadenasso. (2001). Effects of plant invasions on the species richness of abandoned agricultural land. Ecography 24:633–644.

Milly, P. C. D., and Dunne, K. A. (2020). Colorado River flow dwindles as warming-driven loss of reflective snow energizes evaporation. Science, 367(6483), 1252–1255. https://doi.org/10.1126/SCIENCE.AAX0194

Morse, L. E., J. M. Randall, N. Benton, R. Hiebert, and S. Lu. (2004). An invasive species assessment protocol: Evaluating non-native plants for their impact on biodiversity. Version 1. U.S. Government Documents, Utah Regional Depository.

Mote, P. W., Li, S., Lettenmaier, D. P., Xiao, M., and Engel, R. (2018). Dramatic declines in snowpack in the western US. Npj Climate and Atmospheric Science 2018 1:1, 1(1), 1–6. https://doi.org/10.1038/s41612-018-0012-1

Musselman, K.N., Addor, N., Vano, J.A. and Molotch, N.P., (2021). Winter melt trends portend widespread declines in snow water resources. Nature Climate Change, 11(5), pp.418-424.

National Agriculture Imagery Program (NAIP) (2021). USGS EROS Aerial Photography - National Agriculture Imagery Program (NAIP). Digital Object Identifier (DOI) number: /10.5066/F7QN651G. https://www.usgs.gov/centers/eros/science/usgs-eros-archive-aerial-photography-national-agriculture-imagery-program-naip. Accessed September 17, 2024.

National Park Service (NPS). (2007). Final Elk and Vegetation Management Plan/Environmental Impact Statement for Rocky Mountain National Park, Colorado. U.S. Department of the Interior, National Park Service. https://irma.nps.gov/DataStore/Reference/Profile/2237926. Accessed September 17, 2024.

Natural Resources Conservation Service (NRCS). (2017). Soil survey manual. USDA handbook 18, pp.120–131.

Natural Resources Conservation Service (NRCS). (2021). Soil Survey Geographic (SSURGO) Database. Available online at https://sdmdataaccess.sc.egov.usda.gov.

Oksanen J, Simpson G, Blanchet F, Kindt R, Legendre P, Minchin P, O'Hara R, Solymos P, Stevens M, Szoecs E, Wagner H, Barbour M, Bedward M, Bolker B, Borcard D, Carvalho G, Chirico M, De Caceres M, Durand S, Evangelista H, FitzJohn R, Friendly M, Furneaux B, Hannigan G, Hill M, Lahti L, McGlinn D, Ouellette M, Ribeiro Cunha E, Smith T, Stier A, Ter Braak C, Weedon J (2024). vegan: Community Ecology Package_. R package version 2.6-6.1, <https://CRAN.R-project.org/package=vegan>.

Oksanen J., Blanchet F.G., Friendly M., Kindt R., Legendre P., McGlinn D., Minchin P.R., O'Hara R.B., Simpson G.L., Solymos P., Stevens M.H.H., Szoecs E., and Wagner H. (2022). *vegan: Community Ecology Package*. R package version 2.6-2. https://CRAN.R-project.org/package=vegan

Oldham, K. (2010). Troublesome Elk Herd Management Plan Data Analysis Unit E-8, Game Management Units 18 and 181. Colorado Division of Wildlife. Hot Sulpher Springs, CO. 59p.

Overpeck, J. T. (2013). The challenge of hot drought. Nature, 503(7476), 350–351. https://doi.org/10.1038/503350a

Perovich, C., and Sibold, J. S. (2016). Forest composition change after a mountain pine beetle outbreak, Rocky Mountain National Park, CO, USA. Forest Ecology and Management, 366, 184–192. https://doi.org/10.1016/J.FORECO.2016.02.010

Potter, K.M. and Conkling, B.L. (2016). Forest health monitoring: National status, trends, and analysis 2015 (Vol. 213). Government Printing Office.

Pysek, P., D. M. Richardson, M. Rejmanek, G. L. Webster, M. Williamson, and J. Kirschner. (2004). Alien plants in checklists and floras: towards better communication between taxonomists and ecologists. Taxon 53:131–143.

R Core Team (2024). R: A Language and Environment for Statistical Computing_. R Foundation for Statistical Computing, Vienna, Austria. <https://www.R-project.org/>.

Stephenson, N. (1998). Actual evapotranspiration and deficit: biologically meaningful correlates of vegetation distribution across spatial scales. Journal of biogeography, 25(5), 855-870.

Thoma, D. P., Tercek, M. T., Schweiger, E. W., Munson, S. M., Gross, J. E., and Olliff, S. T. (2020). Water balance as an indicator of natural resource condition: Case studies from Great Sand Dunes National Park and Preserve. Global Ecology and Conservation, 24, e01300.

Thornton, P. E., R. Shrestha, M. Thornton, S.-C. Kao, Y. Wei, and B. E. Wilson. (2021). Gridded daily weather data for North America with comprehensive uncertainty quantification. Scientific Data 8. https://doi.org/10.1038/s41597-021-00973-0

Udall, B., and Overpeck, J. (2017). The twenty-first century Colorado River hot drought and implications for the future. Water Resources Research, 53(3), 2404–2418. https://doi.org/10.1002/2016WR019638

Venables, W., and B. Ripley. (2002). Modern applied statistics with S. Fourth edition. Springer, New York. http://www. stats.ox.ac.uk/pub/MASS4.

Westbrook, C., Cooper, D. J., and Baker B. (2006). Beaver dams and overbank floods influence groundwater-surface water interactions of a Rocky Mountain riparian area. Water Resources Research 42: W06404, doi:10.1029/2005WR004560

Wilcox, R., Peterson, T.J. and McNitt-Gray, J.L. (2018). Data analyses when sample sizes are small: Modern advances for dealing with outliers, skewed distributions, and heteroscedasticity. Journal of Applied Biomechanics, 34(4), pp.258-261.

Wilhelm, G., and D. Ladd. (1988). Natural area assessment in the Chicago region. Transactions of the North American Wildlife and Natural Resources Conference 53:361–375.

Williams, J., Stella, J. C., Voelker, S. L., Lambert, A. M., Pelletier, L. M., Drake, J. E., Friedman, J. M., Roberts, D. A., and Singer, M. B. (2022). Local groundwater decline exacerbates response of dryland riparian woodlands to climatic drought. Global Change Biology, 28(22), 6771–6788. https://doi.org/10.1111/GCB.16376
